# Cognitive impairment and dementia—Are they linked to childhood health and socioeconomic status? A systematic review

**DOI:** 10.1371/journal.pone.0311074

**Published:** 2025-03-27

**Authors:** Tung Le, Asri Maharani, Mark Hayter, James Gilleen, Amanda Lee

**Affiliations:** 1 Mental Health Research Group, Division of Nursing, Midwifery and Social Work, Faculty of Biology, Medicine and Health, School of Health Sciences, The University of Manchester, Manchester, United Kingdom; 2 Faculty of Health and Education, School of Nursing and Public Health, Manchester Metropolitan University, Manchester, United Kingdom; Centre d’Estudis Demogràfics: Centre d'Estudis Demografics, SPAIN

## Abstract

**Background:**

Dementia is a major public health concern, with its incidence rising as the population ages. Recent studies suggest links between childhood health, socioeconomic status, and later-life cognitive impairment and dementia, though findings remain inconclusive. This systematic review evaluates the influence of childhood health and socioeconomic status on cognitive impairment and dementia.

**Method and findings:**

A systematic search conducted in MEDLINE, CiNAHL, and PsycINFO in December 2024 identified 44 studies matching our inclusion criteria. Findings are presented under five key themes: (1) childhood health, (2) childhood educational attainment, (3) family socioeconomic and educational factors, (4) childhood experiences, and (5) childhood reading habits and social interactions.

**Conclusion:**

Our results highlight the need for further longitudinal studies to establish causal relationships between early-life risk factors and later cognitive decline. Policymakers should prioritize early childhood development programs that integrate health, nutrition, education, and social support to help mitigate cognitive impairment and dementia in later life.

## Introduction

Our aging population will pass 2 billion by 2050 [1], meaning we face urgent challenges to mitigate age-related diseases, particularly Alzheimer’s disease and other dementias [2]. Dementia care has become a top public health priority due to its widespread prevalence and lack of curative treatments [3]. Consequently, understanding causation is crucial to underpin targeted early interventions and diagnoses. It can inform policy and healthcare strategies that may mitigate the significant burden of dementia and reinforce the disease as a public health priority.

The Lancet Commission on Dementia Prevention, Intervention, and Care identified 14 modifiable risk factors, including childhood-related factors such as low education, which account for 40% of global dementia cases [3]. Lifecourse preventative strategies are supported by the World Health Organization (WHO) 2019 report, “Reducing the Risk of Cognitive Decline and Dementia” [4]. Their guidelines highlight the impact of early-life interventions in mitigating cognitive decline and reinforce a need to evaluate childhood health and socioeconomic conditions as potential contributors to later-life cognitive decline. Thus, this systematic review seeks to reveal early-life risk factors linked to cognitive impairment and dementia.

Malnutrition affects over 149 million children under five and has significant long-term neurodevelopmental consequences [5]. Over 356 million children are living in extreme poverty and lacking access to basic healthcare, food, education, and resources [6]. There is evidence linking poor health or adverse socioeconomic conditions in childhood with later-stage cognitive decline, suggesting those from poorer backgrounds had a 1.5 to 2.0 times higher risk of cognitive decline and dementia [7]. The evidence is clear that childhood nutrition is critical to neurological development, and malnutrition leads to long-term deficits which increase the risk of dementia later in life [8]. Other contributing clinical factors, such as inflammatory defense mechanisms associated with repeated childhood chronic illnesses, and adverse childhood experiences (ACE) are also linked to neurodegeneration and cognitive decline [9,10]. Poor living conditions and limited access to healthcare in children from lower socioeconomic backgrounds mean they are more likely to suffer repeated illnesses [11]. As socioeconomic status (SES) increases, so does access to education and stimulation. Increasingly educated parents provide more learning resources, healthier environments, and greater cognitive stimulation at home, which are linked to improved cognitive function and a reduced risk of dementia in later life [12]. Thus, we can establish that childhood health and SES are factors that pose a long-lasting impact on cognitive health and neurodevelopment.

However, establishing causative links between childhood health, childhood SES, and cognitive function in later life remains contentious. Some studies suggest that the negative effects of poor health or low SES in childhood may be mitigated by positive experiences in adulthood [13,14]. Children may reach higher educational attainment or secure more stable and engaging employment, thus mitigating some of the causative relationships [13,14]. Others oppose this, stating childhood disadvantages have long-term effects on neurodevelopmental structure and function [15], which cannot be entirely mitigated by improvements in later life [16].

In the last few decades, only one systematic review and meta-analysis on early-life factors associated with dementia and cognitive impairment in later life has been conducted [17]. However, epidemiological data in this study spanned from 1865 to 2017, perhaps lacking temporal relevance to today’s society. Search strings were slightly limited for systematic and relevant evidence retrieval, and researcher funding potentially led to intrinsic bias. Thus, our systematic review evaluates current empirical evidence, published within the last decade (2014-2024). Our search strategy also included childhood health and childhood SES to ensure a more comprehensive analysis of early-life factors.

### Definition of childhood health and childhood SES

Childhood health refers to the physical, mental, and emotional well-being of children from birth through adolescence [18]. In the context of this study, childhood health is defined in various ways, ranging from general assessments to more specific measurements. Most studies use retrospective self-reports, asking participants to rate their childhood health from excellent to poor. Additionally, childhood health can be assessed through factors like hunger or food insecurity, as well as the occurrence of illnesses, such as infections or psychological issues, during childhood.

Childhood SES refers to the economic and social conditions experienced during early life, often assessed through a combination of self-reported and objective measures [19]. Common measurements include retrospective self-assessments of social status during childhood, typically using a Likert scale (e.g., high, middle, low), as well as parental education and occupation, which are key indicators of a household’s socioeconomic position. Additional variables include household income, financial stability, and community factors, such as the percentage of adults with higher education or in professional occupations. These measures are typically categorized into “high,” “middle,” or “low” SES and are consistent across studies, although specific variables may vary slightly.

### Research question and objectives

This systematic literature review poses the following research question: “Are there any childhood health and childhood SES factors that may predispose or be linked to cognitive impairment and dementia in later life?”

There are two objectives:

1. To systematically identify and synthesize evidence on the relationship between childhood health and SES with cognitive impairment and dementia in later life.
2. To explore the possible association between these early-life factors and the risk of cognitive impairment and dementia, thereby informing future cohort analyses and intervention strategies.

## Methods

### Design

This study adheres to the reporting standards of the Equator Network for systematic reviews [20], including the implementation of appropriate search strategies and the formulation of research questions based on the structured PICOS framework [21–23]. The eligibility and exclusion criteria for this study are presented in Table 1.

**Table 1:** Eligibility and exclusion criteria using the PICOS framework.

	Criteria
**Population**	Older adults who are at risk for or diagnosed with cognitive impairment or dementia.
**Interest**	The influence of childhood health and socioeconomic status (SES) on cognitive decline and dementia risk in later life.
**Comparison**	Comparisons between participants with diagnosed dementia or cognitive impairment and those without such diagnoses.
**Outcome**	The association of childhood health and SES with dementia and cognitive impairment in later life.
**Study Type**	Includes qualitative studies, quantitative studies, mixed methods studies, and cohort studies published in English, in peer-reviewed journals from 2014 to 2024. Excludes studies with animal subjects, government or organisational reports, books or book chapters, conference abstracts or proceedings, dissertations, theses, commentaries, editorials, and letters.

Briefly, this review focused on studies examining the impact of childhood health and SES on cognitive decline and dementia in older adults, including peer-reviewed research published in the past decade (2014–2024) to ensure the information is up to date.

### Search strategy

We applied the 2024 Medical Subject Headings (MeSH), Boolean operands, and truncations to source relevant studies for this systematic review. The search was conducted in three electronic databases, i.e., MEDLINE, CiNAHL, and PsycINFO, on 17^th^ December 2024. The search strings were:

- MEDLINE: (“child* health*”[All Fields] OR “child* experience*”[All Fields] OR “child* illness”[All Fields] OR “child* disease*”[All Fields] OR “child* socioeconomic”[All Fields] OR “parental education”[All Fields] OR “parental occupation”[All Fields] OR “household income”[All Fields]) AND (“cogn* impairment”[All Fields] OR “cogn* decline”[All Fields] OR “cogn* disorder”[All Fields] OR “dementia”[All Fields] OR “Alzheimer*”[All Fields]) AND (“older adults”[All Fields] OR “older age”[All Fields] OR “older people”[All Fields] OR “elderly”[All Fields] OR “aged”[All Fields] OR “aging”[All Fields]), Filters: in the last 10 years, English.
- CiNAHL: childhood health OR childhood health issues OR childhood socioeconomic status OR (childhood adversity or childhood trauma or adverse childhood experiences) AND (cognitive impairment or cognitive dysfunction or cognitively impaired or dementia or Alzheimer) AND (older people or older adults or elderly or aged), Filter: Publication Year: 2014-2024, Peer-Reviewed, English Language, Human.
- PsycINFO: Any Field: child * health * OR Any Field: child * experience * OR Any Field: child * illness OR Any Field: child * disease * OR Any Field: child * socioeconomic OR Any Field: parental education OR Any Field: parental occupation OR Any Field: household income AND Any Field: cogn * impairment OR Any Field: cogn * decline OR Any Field: dementia OR Any Field: Alzheimer * AND Population Group: Human AND Age Group: Middle Age (40-64 yrs) OR Aged (65 yrs & older) AND Document Type: Journal Article AND Open Access AND Peer-Reviewed Journals only AND Year: 2014 To 2024.

We also conducted a manual search of the reference lists of relevant articles to identify additional studies. Following Equator guidelines, all authors meticulously reviewed the references of all included studies to eliminate duplicates and exclude articles that did not align with the scope of the study. The search was restricted to articles published in English. Duplicates were manually removed after identifying high-risk duplicate articles using the Rayyan platform. TL and AM independently assessed eligibility, and any disagreements were resolved by the final decision from AL.

### Study selection

**Fig 1:**
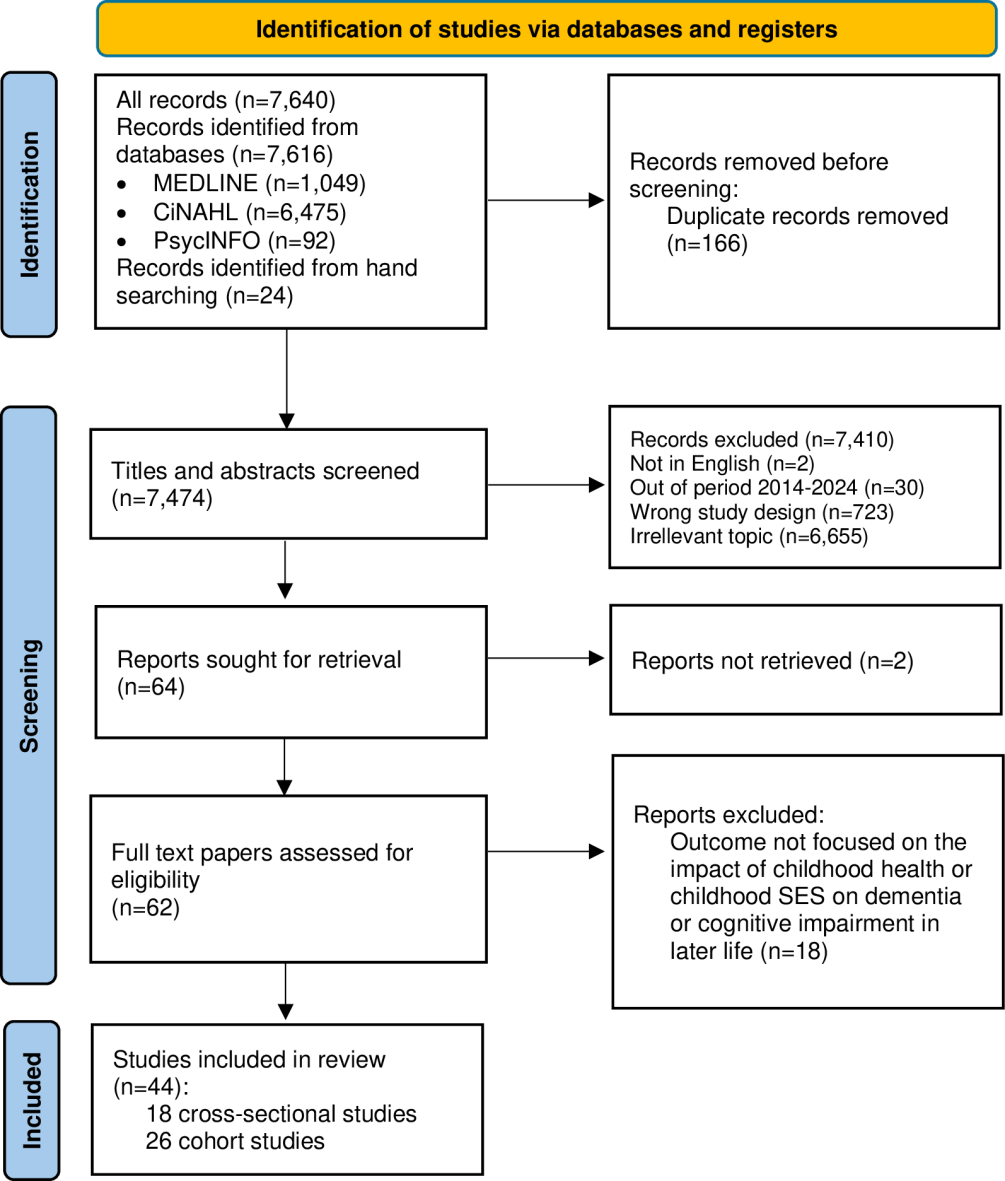
PRISMA flow diagram.

The search strategy identified 7,616 records from electronic databases (1049 articles from MEDLINE, 6475 articles from CINAHL, and 92 articles from PsycINFO) and 24 from hand searching, resulting in 7,640 publications for initial evaluation. After removing 166 duplicates, 7,474 records were screened by title and abstract, resulting in the exclusion of 7,410 records for reasons including: not in English, was not published from 2014 to 2024, wrong study design, and irrelevant topic. Of the remaining 64 reports sought for retrieval, 2 were not retrieved, leaving 62 full-text papers to be assessed for eligibility. A further 18 reports were excluded because the outcome was not focused on exploring the impact of childhood health or childhood SES on dementia or cognitive impairment in later life. Ultimately, 44 publications were selected as the most relevant for the systematic review, comprising 18 cross-sectional studies and 26 cohort studies (Fig 1).

### Quality appraisal

To assess article quality for this literature review, we utilized the Joanna Briggs Institute (JBI) tool, known for its superior reliability compared to the Critical Appraisal Checklists Programme (CASP) [24]. The JBI checklist comprises eight questions for cross-sectional studies and 11 questions for cohort studies (See Tables 2 and 3 for results of quality appraisal) [25]. Responses were categorized as “yes” (1 point) and others as 0 points (“no,” “unclear,” or “not applicable”). Tertile classifications of high- (score > 70%), mid- (score 50–70%), and low-quality (score < 50%) were assigned using the appropriate JBI checklist. Only “high-quality” papers were included in the review. Quality assessments of each study were conducted independently by TL and AM, with cross-checking by AL to minimize bias and ensure that no data were missing in this systematic review.

**Table 2:** JBI quality assessment for cross-sectional studies.

No	Authors	Title	Q1	Q2	Q3	Q4	Q5	Q6	Q7	Q8	Score (%)	Classify
**1**	Lee et al. (2023) [26]	Adverse Childhood Experiences and Aging‐Associated Functional Impairment in a National Sample of Older Community‐Dwelling Adults	Yes	Yes	Yes	Yes	No	No	Yes	Yes	75	High quality
**2**	Brown et al. (2022) [27]	Adverse Childhood Experiences and Subjective Cognitive Decline in the US	Yes	Yes	Yes	Yes	Yes	Yes	Yes	Yes	100	High quality
**3**	Gold et al. (2021) [28]	Are adverse childhood experiences associated with late-life cognitive performance across racial/ethnic groups: results from the Kaiser Healthy Aging and Diverse Life Experiences study baseline	Yes	Yes	Yes	Yes	Yes	Unclear	Yes	Yes	87.5	High quality
**4**	Halpin et al. (2022) [29]	Are Adverse Childhood Experiences Associated with Worse Cognitive Function in Older Adults?	Yes	Yes	Yes	Yes	Yes	Unclear	Yes	Yes	87.5	High quality
**5**	Morita and Fujiwara (2021) [30]	Association between childhood parental involvement and late-life cognitive function: A population-based cross- sectional study among cognitively intact community-dwelling older adults in Japan	Yes	Yes	Yes	Yes	Yes	Yes	Yes	Yes	100	High quality
**6**	Nishizawa et al. (2019) [31]	Association between childhood socioeconomic status and subjective memory complaints among older adults: results from the Japan Gerontological Evaluation Study 2010	Yes	Yes	Yes	Yes	Yes	Yes	Yes	Yes	100	High quality
**7**	Baiden et al. (2022) [32]	Association of adverse childhood experiences with subjective cognitive decline in adulthood: Findings from a population-based study	Yes	Yes	Yes	Yes	Yes	Unclear	Yes	Yes	87.5	High quality
**8**	Filigrana et al. (2023) [33]	Childhood and Life-Course Socioeconomic Position and Cognitive Function in the Adult Population of the Hispanic Community Health Study of Latinos	Yes	Yes	Yes	Yes	Yes	Yes	Yes	Yes	100	High quality
**9**	Kobayashi et al. (2017) [34]	Childhood deprivation and later-life cognitive function in a population-based study of older rural South Africans	Yes	Yes	Yes	Yes	Yes	Yes	Yes	Yes	100	High quality
**10**	Muhammad et al. (2022) [35]	Childhood deprivations predict late‐life cognitive impairment among older adults in India	Yes	Yes	Yes	Yes	Yes	Yes	Yes	Yes	100	High quality
**11**	Rotstein and Levine (2021) [36]	Childhood infectious diseases and old age cognitive functioning: a nationally representative sample of community-dwelling older adults	Yes	Yes	Yes	Yes	Yes	Yes	Yes	Yes	100	High quality
**12**	Maharani (2019) [37]	Childhood Socioeconomic Status and Cognitive Function Later in Life: Evidence From a National Survey in Indonesia	Yes	Yes	Yes	Yes	Yes	Yes	Yes	Yes	100	High quality
**13**	Korinek et al. (2024) [38]	Cognitive function following early life war-time stress exposure in a cohort of Vietnamese older adults	Yes	Yes	Yes	Yes	Yes	No	Yes	Yes	87.5	High quality
**14**	Momtaz et al. (2015) [39]	Does food insufficiency in childhood contribute to dementia in later life?	Yes	Yes	Yes	Yes	Yes	Yes	Yes	Yes	100	High quality
**15**	Voyer et al. (2023) [40]	Linking Adverse Childhood Experiences and Other Risk Factors to Subjective Cognitive Decline in an Aging Population	Yes	Yes	Yes	Yes	Yes	Yes	Yes	Yes	100	High quality
**16**	Lian et al. (2024) [41]	No Association Found- Adverse Childhood Experiences and Cognitive Impairment in Older Australian Adults	Yes	Yes	Yes	Yes	Yes	Yes	Yes	Yes	100	High quality
**17**	Nilaweera et al. (2022) [42]	The association between adverse childhood events and later-life cognitive function and dementia risk	Yes	Yes	Yes	Yes	Yes	Yes	Yes	Yes	100	High quality
**18**	Schickedanz et al. (2022) [43]	The Association Between Adverse Childhood Experiences and Positive Dementia Screen in American Older Adults	Yes	Yes	Yes	Yes	Yes	Unclear	Yes	Yes	87.5	High quality

*Q1. Were the criteria for inclusion in the sample clearly defined?; Q2. Were the study subjects and the setting described in detail?; Q3. Was the exposure measured in a valid and reliable way?; Q4. Were objective, standard criteria used for measurement of the condition?; Q5. Were confounding factors identified?; Q6. Were strategies to deal with confounding factors stated?; Q7. Were the outcomes measured in a valid and reliable way?; Q8. Was the statistical analysis used appropriate?

**Table 3:** JBI quality assessment for cohort studies.

No	Authors	Title	Q1	Q2	Q3	Q4	Q5	Q6	Q7	Q8	Q9	Q10	Q11	Score (%)	Classify
**1**	Chai et al. (2024) [44]	Association between childhood parental literacy and late-life cognitive function- The Guangzhou Biobank Cohort Study	Yes	Yes	Yes	Yes	No	Yes	Yes	Yes	Yes	No	Yes	81.82	High quality
**2**	Zhou and Zhao (2024) [45]	Childhood Peer Relationships and Dementia Risk in Chinese Older Adults- A Mediation Analysis	Yes	Yes	Yes	Yes	Yes	Yes	Yes	Yes	Yes	Yes	Yes	100	High quality
**3**	He and Yang (2024) [46]	Longitudinal association of adverse childhood experiences with cognitive function trajectories among middle-aged and older adults- group-based trajectory modeling	Yes	Yes	Yes	Yes	No	Yes	Yes	Yes	Yes	Yes	Yes	90.91	High quality
**4**	Ma et al. (2021) [47]	The influence of childhood adversities on mid to late cognitive function: From the perspective of life course	Yes	Yes	Yes	Yes	Unclear	Yes	Yes	Yes	Yes	No	Yes	81.82	High quality
**5**	Yang and Wang (2020) [48]	Early-Life Conditions and Cognitive Function in Middle-and Old-Aged Chinese Adults: A Longitudinal Study	Yes	Yes	Yes	Yes	No	Yes	Yes	Yes	Yes	Yes	Yes	100	High quality
**6**	Sha et al. (2018) [49]	Associations of childhood socioeconomic status with mid‐life and late‐life cognition in Chinese middle‐aged and older population based on a 5‐year period cohort study	Yes	Yes	Yes	No	No	Yes	Yes	Yes	Yes	No	Yes	72.73	High quality
**7**	Lin et al. (2022) [50]	Association of Adverse Childhood Experiences and Social Isolation With Later-Life Cognitive Function Among Adults in China	Yes	Yes	Yes	Yes	Yes	Yes	Yes	Yes	Yes	Yes	Yes	100	High quality
**8**	Yuan et al. (2022) [51]	Heterogeneous adverse childhood experiences and cognitive function in an elderly Chinese population: a cohort study	Yes	Yes	Yes	Yes	Yes	Yes	Yes	Yes	Yes	No	Yes	90.91	High quality
**9**	Aartsen et al., (2019) [52]	Advantaged socioeconomic conditions in childhood are associated with higher cognitive functioning but stronger cognitive decline in older age	Yes	Yes	Yes	Yes	Yes	Yes	Yes	Yes	Yes	Yes	Yes	100	High quality
**10**	Lewis et al. (2023) [53]	Availability of Cognitive Resources in Early Life Predicts Transitions Between Cognitive States in Middle and Older Adults From Europe	Yes	Yes	Yes	Yes	Unclear	Yes	Yes	Yes	Yes	Yes	Yes	90.91	High quality
**11**	Donley et al. (2018) [54]	Association of childhood stress with late-life dementia and Alzheimer’s disease: the KIHD study	Yes	Yes	Yes	Yes	Yes	Yes	Yes	Yes	Yes	No	Yes	90.91	High quality
**12**	Tani et al. (2021) [55]	Adverse Childhood Experiences and Dementia: Interactions With Social Capital in the Japan Gerontological Evaluation Study Cohort	Yes	Yes	Yes	Yes	Yes	Yes	Yes	Yes	Yes	No	Yes	90.91	High quality
**13**	Tani et al. (2020) [56]	Association Between Adverse Childhood Experiences and Dementia in Older Japanese Adults	Yes	Yes	Yes	Yes	Yes	Yes	Yes	Yes	Yes	No	Yes	90.91	High quality
**14**	Gutierrez et al. (2024) [57]	My Parent, Myself, or My Child- Whose Education Matters Most for Trajectories of Cognitive Aging in Middle Age	Yes	Yes	Yes	Yes	Yes	Yes	Yes	Yes	Yes	Yes	Yes	100	High quality
**15**	Hendrie et al. (2018) [58]	The Association of Early Life Factors and Declining Incidence Rates of Dementia in an Elderly Population of African Americans	Yes	Yes	Yes	No	No	Yes	Yes	Yes	Yes	No	Yes	72.73	High quality
**16**	Racine Maurice et al. (2021) [59]	Childhood Socioeconomic Status Does Not Predict Late-Life Cognitive Decline in the 1936 Lothian Birth Cohort	Yes	Yes	Yes	Yes	No	Yes	Yes	Yes	Yes	Unclear	Yes	81.82	High quality
**17**	Dekhtyar et al. (2015) [60]	A Life-Course Study of Cognitive Reserve in Dementia--From Childhood to Old Age	Yes	Yes	Yes	Yes	Yes	Yes	Yes	Yes	Yes	No	Yes	90.91	High quality
**18**	Künzi et al. (2024) [61]	The impact of early adversity on later life health, lifestyle, and cognition	Yes	Yes	Yes	Yes	No	Yes	Yes	Yes	Yes	Yes	Yes	90.91	High quality
**19**	Tsang et al. (2022) [62]	The long arm of childhood socioeconomic deprivation on mid- to later-life cognitive trajectories: A cross-cohort analysis	Yes	Yes	Yes	Yes	Yes	Yes	Yes	Yes	Yes	Yes	Yes	100	High quality
**20**	Thomas et al. (2024) [63]	Early-Life Parental Affection, Social Relationships in Adulthood, and Later-Life Cognitive Function	Yes	Yes	Yes	Yes	Yes	Yes	Yes	Yes	Yes	Yes	Yes	100	High quality
**21**	Lor et al. (2023) (29) [64]	What is the association between adverse childhood experiences and late-life cognitive decline? Study of Healthy Aging in African Americans (STAR) cohort study	Yes	Yes	Yes	Yes	Yes	Yes	Yes	Yes	Yes	No	Yes	90.91	High quality
**22**	Kucharska-Newton et al. (2023) [65]	Association of Childhood and Midlife Neighborhood Socioeconomic Position With Cognitive Decline	Yes	Yes	Yes	Yes	Yes	Yes	Yes	Yes	Yes	Unclear	Yes	90.91	High quality
**23**	Greenfield et al. (2021) [66]	Life Course Pathways From Childhood Socioeconomic Status to Later-Life Cognition: Evidence From the Wisconsin Longitudinal Study	Yes	Yes	Yes	Yes	Yes	Yes	Yes	Yes	Yes	Yes	Yes	100	High quality
**24**	Lee et al. (2021) [67]	Multigenerational Households During Childhood and Trajectories of Cognitive Functioning Among U.S. Older Adults	Yes	Yes	Yes	No	No	Yes	Yes	Yes	Yes	No	Yes	72.73	High quality
**25**	Tom et al. (2020) [68]	Association of Demographic and Early-Life Socioeconomic Factors by Birth Cohort With Dementia Incidence Among US Adults Born Between 1893 and 1949	Yes	Yes	Yes	Unclear	No	Yes	Yes	Yes	Yes	Yes	Yes	81.82	High quality
**26**	Zhang et al. (2020) [69]	Early-life socioeconomic status, adolescent cognitive ability, and cognition T in late midlife: Evidence from the Wisconsin Longitudinal Study	Yes	Yes	Yes	No	Unclear	Yes	Yes	Yes	Yes	No	Yes	72.73	High quality

*Q1. Were the two groups similar and recruited from the same population?; Q2. Were the exposures measured similarly to assign people to both exposed and unexposed groups?; Q3. Was the exposure measured in a valid and reliable way?; Q4. Were confounding factors identified?; Q5. Were strategies to deal with confounding factors stated?; Q6. Were the groups/participants free of the outcome at the start of the study (or at the moment of exposure)?; Q7. Were the outcomes measured in a valid and reliable way?; Q8. Was the follow up time reported and sufficient to be long enough for outcomes to occur?; Q9. Was follow up complete, and if not, were the reasons to loss to follow up described and explored?; Q10. Were strategies to address incomplete follow up utilized?; Q11. Was appropriate statistical analysis used?

### Data extraction

We extracted data from 44 articles using an Excel spreadsheet. Table 4 presents the detailed empirical research matrix: (1) Authors; (2) Title; (3) Country/Region; (4) Study design; (5) Data source; (6) Age of participants; (7) Sample size; (8) Data analysis methods; (9) Early-life factors; (10) Cognitive/dementia measures; and (11) Main outcomes.

**Table 4:** Empirical research matrix presenting studies included in the systematic review (N = 44).

No	Authors	Title	Country/Region	Study design	Data source	Age of participants	Sample size	Data analysis methods	Early-life factors	Cognitive/dementia measures	Main outcomes
**1**	Dekhtyar et al. (2015) [60]	A Life-Course Study of Cognitive Reserve in Dementia--From Childhood to Old Age	Sweden	Cohort	The Uppsala Birth Cohort Multigeneration Study 1915-1929	65+	7,574	Descriptive statistics, discrete time proportional hazard models	Education	Dementia diagnosis in Medical record	The risk of dementia was lower among those with higher childhood school grades (hazard ratio [HR]: 0.79; 95% confidence interval [CI]: 0.68 to 0.93).
**2**	Aartsen et al. (2019) [52]	Advantaged socioeconomic conditions in childhood are associated with higher cognitive functioning but stronger cognitive decline in older age	Europe	Cohort	The Survey of Health, Ageing, and Retirement in Europe (SHARE)	50-96	24,066	Descriptive statistics, linear and non- linear mixed-effect models	Childhood SES	Delayed recall and verbal fluency	Compared to those with the most disadvantaged childhood conditions, those in the most advantaged socioeconomic positions scored 1.27 more words for delayed recall and 5.39 more words for verbal fluency at age 73.
**3**	Lee et al. (2023) [26]	Adverse Childhood Experiences and Aging‐Associated Functional Impairment in a National Sample of Older Community‐Dwelling Adults	US	Cross-sectional	The National Social Life, Health and Aging Project (NSHAP)	50+	3,387	Descriptive statistics, Chi-square tests, multivariable logistic regression models	Physical violence or abuse, witnessing of physical violence or abuse, family financial stress, separation from a parent, poor childhood health, and unhappy family life	Montreal Cognitive Assessment (MoCA-SA)	Participants were more likely to demonstrate at least mild cognitive impairment if they reported a history of poor or fair childhood health (OR 1.74, 95% CI 1.20–2.53). After adjusting for age, gender, race, and ethnicity, participants reporting any ACE history were more likely to demonstrate physical mobility impairment (OR 1.30, 95% CI 1.11–1.52) and cognitive impairment (OR 1.26, 95% CI 1.03–1.54) and report functional disability (OR 1.69, 95% CI 1.38–2.07), compared to those with no ACE history
**4**	Tani et al. (2021) [55]	Adverse Childhood Experiences and Dementia: Interactions With Social Capital in the Japan Gerontological Evaluation Study Cohort	Japan	Cohort	The Japan Gerontological Evaluation Study 2013-2016	65+	16,821	Descriptive statistics, Cox regression models	Parental loss or parental divorce, family psychopathology, abuse and neglect	Mini-Mental State Examination	Those with more ACEs had a greater risk of dementia. The hazard ratio for ≥ 3 ACEs was 3.25 (95% CI = 1.73–6.10) among those with low social capital and 1.19 (95% CI = 0.58–2.43) among those with middle social capital. ACEs were associated with increased dementia incidence only for those with low social capital.
**5**	Brown et al. (2022) [27]	Adverse Childhood Experiences and Subjective Cognitive Decline in the US	US	Cross-sectional	The 2019 Behavioral Risk Factor Surveillance System Survey (BRFSS)	45+	82,688	Descriptive statistics, multivariable logistic regression	ACEs included sexual, physical/psychological and environmental ACEs	Confusion and memory loss	Sexual (adjusted OR (aOR: 2.83; 95% CI: 2.42 – 3.31)), physical/psychological (aOR: 2.05; 95% CI: 1.83 – 2.29) and environmental (aOR: 1.94; 95% CI: 1.74 – 2.16) ACEs were associated with SCD. There was also a dose-response relationship between ACE score and SCD
**6**	Gold et al. (2021) [28]	Are adverse childhood experiences associated with late-life cognitive performance across racial/ethnic groups: results from the Kaiser Healthy Aging and Diverse Life Experiences study baseline	US	Cross-sectional	Kaiser Healthy Aging and Diverse Life Experiences Study 2017	65+	1,661	Descriptive statistics, covariate-adjusted mixed-effects linear regression models	Parents’ education, divorce, remarriage, domestic violence, witnessing substance abuse, parent’s job loss, parental incarceration, family member’s illness, and death of mother or father	Verbal episodic memory, semantic memory and executive functioning	Parent’s remarriage (β = − 0.11; 95% CI: − 0.20 to − 0.03), mother’s death (β = − 0.18; 95% CI: − 0.30 to − 0.07), and father’s death (β = − 0.11; 95% CI: − 0.20 to − 0.01) were associated with worse cognition.
**7**	Halpin et al. (2022) [29]	Are Adverse Childhood Experiences Associated with Worse Cognitive Function in Older Adults?	US	Cross-sectional	The Maine-Aging Behavior Learning Enrichment (M-ABLE) Study at the University of Maine	55-90	121	Descriptive statistics, Spearman’s, Pearson, hierarchical regressions	Childhood experiences of abuse, neglect, and household dysfunction	The National Institute of Health-Toolbox for the Assessment of Neurological and Behavioral Function Quality of Life in Neurological Disorders (NIH-TB Neuro-QOL, Version 2.0)	ACE scores were negatively associated with income and years of education and positively associated with depressive symptoms and SCC. ACE scores were a significant predictor of intellectual function and executive attention; however, these relationships were no longer significant after adjusting for education
**8**	Tani et al. (2020) [56]	Association Between Adverse Childhood Experiences and Dementia in Older Japanese Adults	Japan	Cohort	The Japan Gerontological Evaluation Study 2013-2016	65+	17,412	Descriptive statistics, Cox regression models	Parental loss or parental divorce, family psychopathology, and abuse and neglect	Mini-Mental State Examination	Participants who experienced 3 or more ACEs had a greater risk of developing dementia compared to those without ACEs (HR = 1.78; 95% CI: 1.15–2.75; P = 0.009).
**9**	Morita and Fujiwara (2021) [30]	Association between childhood parental involvement and late-life cognitive function: A population-based cross- sectional study among cognitively intact community-dwelling older adults in Japan	Japan	Cross-sectional	Self-administered survey	65-88	266	Descriptive statistics, multiple regression analysis models	Parents often read books, helped with homework, discussed issues, went on outings, played outside, and cooked together	Quick Mild Cognitive Impairment	Participants with greater positive parental involvement in childhood showed higher total QMCI scores (P < 0.001). Those with low, medium, and high parental involvement had higher scores by 2.30 (95% CI: 0.36–4.24), 2.83 (95% CI: 0.41–5.23), and 6.00 (95% CI: 2.39–9.61) points, respectively, compared to those without positive parental involvement. Book reading showed a significant positive association with the total score.
**10**	Chai et al. (2024) [44]	Association between childhood parental literacy and late-life cognitive function- The Guangzhou Biobank Cohort Study	China	Cohort	The Guangzhou Health and Happiness Association for the Respectable Elders (GHHARE)	50+	8,891	Descriptive statistics, ANOVA and chi-square test, multivariable linear regression, logistic regression	Parental literacy	Mini-Mental State Examination and Delayed Word Recall Test	Compared with those whose childhood parents could not read/ write, those with only the father, only the mother, or both parents able to read/write during childhood had higher scores on the MMSE and its dimensions, and lower odds of mild cognitive impairment (MCI)
**11**	Nishizawa et al. (2019) [31]	Association between childhood socioeconomic status and subjective memory complaints among older adults: results from the Japan Gerontological Evaluation Study 2010	Japan	Cross-sectional	The Japan Gerontological Evaluation Study 2010-2011	65+	16,184	Descriptive statistics, Poisson regression models	Childhood SES	Subjective memory complaints	Compared with those who experienced high SES during childhood, those with low SES were 1.29 times (95% CI: 1.22–1.36) and those with middle SES were 1.10 times (95% CI: 1.04–1.17) more likely to show subjective memory complaints.
**12**	Lin et al. (2022) [50]	Association of Adverse Childhood Experiences and Social Isolation With Later-Life Cognitive Function Among Adults in China	China	Cohort	The China Health and Retirement Longitudinal Study Wave 1,2,3 2011-2015	45-97	6,466	Descriptive statistics, linear mixed-effects models	ACEs (i.e., physical abuse, household substance abuse, domestic violence, unsafe neighborhood, and bullying, emotional neglect, household mental illness, incarcerated household member, parental separation or divorce, and parental death)	Episodic memory and executive function	Compared with no exposures, experience of 1 deprivation-related ACE was associated with faster cognitive decline in global cognition (β = − 0.012 [95% CI, − 0.022 to − 0.002] SD/y) and executive function (β = − 0.010 [95% CI, − 0.020 to − 0.00002] SD/y), whereas individuals with at least 2 childhood deprivations had faster cognitive declines in all cognitive tests (β = − 0.035 [95% CI, − 0.050 to − 0.019] SD/y for global cognition; β = − 0.047 [95% CI, − 0.068 to − 0.025] SD/y for episodic memory; β = − 0.019 [95% CI, − 0.034 to − 0.004] SD/y for executive function). However, such an association was not observed for threat-related ACEs.
**13**	Baiden et al. (2022) [32]	Association of adverse childhood experiences with subjective cognitive decline in adulthood: Findings from a population-based study	US	Cross-sectional	The 2019 Behavioral Risk Factor Surveillance System Survey (BRFSS)	45-79	50,277	Descriptive statistics, binary logistic regression	Childhood abuse (i.e., emotional, physical, and sexual) and household dysfunction (i.e., mental illness, substance use, incarceration, separation/divorce, and witnessing domestic violence	Confusion and memory loss	Respondents who had four or more adverse childhood experiences had 2.98 times higher odds of having subjective cognitive decline when compared to respondents with no adverse childhood experiences (aOR = 2.98, 95% CI = 2.56-3.48).
**14**	Kucharska-Newton et al. (2023) [65]	Association of Childhood and Midlife Neighborhood Socioeconomic Position With Cognitive Decline	US	Cohort	The community-based Atherosclerosis Risk in Communities (ARIC) Study	45-64	5711	Descriptive statistics, multinomial linear mixed-effects model	Median household income; median value of owner- occupied housing units; percentage of households receiving interest, dividend, or net rental income; percentage of adults with a high school degree; percentage of adults with a college degree; and percentage of adults in professional, managerial, or executive occupations.	Word Fluency Test, Digit Symbol Substitution Test, and Delayed Word Recall Test, Digit Span Backward Test, Boston Naming Test, Animal Naming Test, Trail Making Tests A and B, Incidental Learning Test, and Logical Memory Test	Each 1-SD-higher childhood SES score was associated with a slower (β, − 9.2%; 95% CI, − 12.1% to − 6.4%) rate of cognitive decline relative to the sample median. A comparable association was observed when comparing the highest tertile with the lowest tertile of childhood SES (β, − 17.7%; 95% CI, − 24.1% to − 11.3%)
**15**	Donley et al. (2018) [54]	Association of childhood stress with late-life dementia and Alzheimer’s disease: the KIHD study	Finland	Cohort	The Kuopio Ischemic Heart Disease Risk Factor Study since 1984	42-61	2,682	Descriptive statistics, multiple covariates analyses	Stress	ICD-8 code 290, ICD-9 codes 4378A and 290, and ICD-10	Childhood stress associated with increased risk of dementia (HR = 1.86, 95% CI: 1.12–3.10). Associations remained statistically significant after adjustment for age, education, income, and other covariates (HR = 1.93, 95% CI: 1.14–3.25).
**16**	Tom et al. (2020) [68]	Association of Demographic and Early-Life Socioeconomic Factors by Birth Cohort With Dementia Incidence Among US Adults Born Between 1893 and 1949	US	Cohort	The Adult Changes in Thought study	65+	4,277	Descriptive statistics, Fine and Gray subdistribution proportional hazards model	Childhood financial stability	The Cognitive Abilities Screening Instrument	Indicators of a more advantaged early-life environment (OR = 0.91, 95% CI 0.84-0.98) were associated with a lower incidence of dementia. Lower educational level (<= high school) (OR = 1.23, 95% CI: 1.07-1.40) were associated with a higher incidence of dementia.
**17**	Sha et al. (2018) [49]	Associations of childhood socioeconomic status with mid‐life and late‐life cognition in Chinese middle‐aged and older population based on a 5‐year period cohort study	China	Cohort	The China Health and Retirement Longitudinal Study Wave 1,2,3 2011-2015	45-90	10,533	Descriptive statistics, latent growth curve models	Family financial status, parent’s education attainments, health	Telephone Interview of Cognitive Status, word recall, and drawing a figure successfully	Cognition in the 45-59 age cohort showed a curvilinear change, while cognition in the 60-90 age cohort showed a linear decline. Participants with higher childhood SES, including higher self-evaluated financial status, higher parental education, and better health, were associated with better mid-life cognitive performance.
**18**	Lewis et al. (2023) [53]	Availability of Cognitive Resources in Early Life Predicts Transitions Between Cognitive States in Middle and Older Adults From Europe	Europe	Cohort	The Survey of Health, Ageing, and Retirement in Europe (SHARE)	60+	32,783	Descriptive statistics, multistate survival models, multinomial regression model	Access to books	Telephone Interview of Cognitive Status	Access to more books at age 10 was associated with a decreased risk of developing cognitive impairment (adjusted hazard ratio = 0.79, confidence interval: 0.76–0.82). Total longevity was similar between participants reporting high (+1 standard deviation [SD]) and low (−1 SD) number of books in the childhood home; however, individuals with more access to childhood books lived a greater proportion of this time without cognitive impairment.
**19**	Filigrana et al. (2023) [33]	Childhood and Life-Course Socioeconomic Position and Cognitive Function in the Adult Population of the Hispanic Community Health Study Study of Latinos	US	Cross-sectional	The Hispanic Community Health Study/Study of Latinos (HCHS/SOL)	45-74	9,331	Descriptive statistics, mediation analysis	Parental education	The Six- Item Screener (SIS), a brief measure of global mental status (34); the 2 scores of the Brief Spanish-English Verbal Learning Test (B-SEVLT) (35), a measure of verbal learning and memory; the Controlled Oral Word Association or Word Fluency Test (WF) (36), a measure of verbal functioning; and the Digit Symbol Substitution Test (DSS) of the Wechsler Adult Intelligence Scale–Revised, a measure of psychomotor speed and sustained attention	High childhood SEP was associated with global cognition in adulthood (coefficient for parental education beyond high school vs. less than high school = 0.26, 95% confidence interval: 0.15, 0.37).
**20**	Kobayashi et al. (2017) [34]	Childhood deprivation and later-life cognitive function in a population-based study of older rural South Africans	South Africa	Cross-sectional	Health and Aging in Africa: Longitudinal Study of an INDEPTH Community since 1992	40+	5,059	Descriptive statistics, linear regression models	Health, education, parent’s occupation	Time orientation, numeracy, and word recall	Poor childhood health was associated with lower cognitive scores (total effect = − 0.28; 95% CI = − 0.35 to − 0.21, compared to good health). Having a father in a professional job during childhood, although rare (3% of the sample), was associated with better cognitive scores (total effect = 0.25; 95% CI = 0.10 to 0.40, compared to unskilled manual labor, which 29% of the sample had). Education was positively and linearly associated with later-life cognitive function (effect = 0.09; 95% CI = 0.09 to 0.10 per additional year of education).
**21**	Muhammad et al. (2022) [35]	Childhood deprivations predict late‐life cognitive impairment among older adults in India	India	Cross-sectional	The Longitudinal Ageing Study in India (LASI) Wave 1 2017-2018	60+	31,464	Descriptive statistics, moderated multiple linear regression models	Childhood health status (good, fair and poor) and childhood SES (good, average, poor)	Mini-Mental State Examination	Older adults who had fair health during childhood were more likely to suffer from cognitive impairment compared to those with good childhood health (Coef: 0.60; CI 0.39–0.81). Those with poor childhood financial status were more likely to suffer from cognitive impairment compared to those with good childhood financial status (Coef: 0.81; CI 0.56–1.07). Older adults with both fair childhood health and poor childhood financial status were more likely to suffer from cognitive impairment compared to those with both good childhood health and good financial status (Coef: 1.26; CI 0.86–1.66).
**22**	Rotstein and Levine (2021) [36]	Childhood infectious diseases and old age cognitive functioning: a nationally representative sample of community-dwelling older adults	Ireland	Cross-sectional	The Irish Longitudinal Study on Ageing (TILDA)	65-85	2,994	Descriptive statistics, linear regression models	Childhood infectious diseases (i.e., chicken pox, measles, and mumps)	Mini-Mental State Examination	The most parsimonious model was a linear adjusted model (Bayesian Information Criterion = 12646.09). Late-life cognitive functioning significantly improved as the number of childhood infectious diseases increased (β = 0.18; 95% CI = 0.11, 0.26; p < 0.001). This effect was not significantly attenuated in all sensitivity analyses.
**23**	Zhou and Zhao (2024) [45]	Childhood Peer Relationships and Dementia Risk in Chinese Older Adults- A Mediation Analysis	China	Cohort	The China Health and Retirement Longitudinal Study Wave 1,2,3,4 2011-2018	60+	7,574	Descriptive statistics, marginal structural models	Childhood Peer Relationships	Telephone Interview for Cognitive Status and its modifications (TICS‐m) 10‐word immediate and delayed recall test for episodic memory (score range: 0–20), a serial 7’s subtraction task for working memory (score range: 0–5), and a backwards counting task for attention (score range: 0–2).	Individuals with deficits in childhood peer relationships had a higher risk of dementia (odds ratio [OR], 1.21; 95% confidence interval [CI], 1.10–1.34) compared with those with more positive experiences.
**24**	Racine Maurice et al. (2021) [59]	Childhood Socioeconomic Status Does Not Predict Late-Life Cognitive Decline in the 1936 Lothian Birth Cohort	Scotland	Cohort	The Lothian Birth Cohort 1936 (LBC1936)	70-82	519	Descriptive statistics, multiple linear regressions	Parental Sociodemographics, Parental Social Class	Mini-Mental State Examination	Participants with less educated mothers showed an increase in cognitive decline (β = − 0.132, p = 0.048, and CI = − 0.80, − 0.00). The relationship between maternal educational attainment and cognitive decline became non-significant when controlling for adult SES (i.e., participant educational attainment and occupation)
**25**	Maharani (2019) [37]	Childhood Socioeconomic Status and Cognitive Function Later in Life: Evidence From a National Survey in Indonesia	Indonesia	Cross-sectional	The Indonesia Family Life Survey (IFLS) Wave 5 2014-2015	50+	6,676	Descriptive statistics, linear regression models	Hunger, the availability of facilities, and the number of books in the home	Telephone Interview for Cognitive Status	Numbers of facilities and books available in childhood homes substantially associated with cognition in later life.
**26**	Korinek et al. (2024) [38]	Cognitive function following early life war-time stress exposure in a cohort of Vietnamese older adults	Vietnam	Cross-sectional	The Vietnam Health and Aging Study	59+	2,447	Descriptive statistics, quantile regression	Severe hunger in childhood and environmental hardships, war stress exposure	Mini-Mental State Examination	Severe childhood hunger and environmental hardships are linked to poorer cognitive function in later life. PTSD, hypertension, and stroke, all exacerbated by wartime stress, are also associated with lower cognitive scores.
**27**	Momtaz et al. (2015) [39]	Does food insufficiency in childhood contribute to dementia in later life?	Malaysia	Cross-sectional	The Mental Health and Quality of Life of Older Malaysians 2003-2005	60+	2,745	Descriptive statistics, multiple binary logistic regression	Food insufficiency	Geriatric Mental State-Automated Geriatric Examination for Computer Assisted Taxonomy	Food insufficiency in childhood independently increased the risk of developing dementia in old age by 81%, after adjusting for sociodemographic factors (odds ratio = 1.81, 95% confidence interval 1.13–2.92, P < 0.01).
**28**	Yang and Wang (2020) [48]	Early-Life Conditions and Cognitive Function in Middle-and Old-Aged Chinese Adults: A Longitudinal Study	China	Cohort	The China Health and Retirement Longitudinal Study Wave 1,2,3 2011-2015	45-101	16,258	Descriptive statistics, multilevel growth curve modeling	Early parental death, childhood SES (i.e., education and occupation of father), food deprivation, and childhood health	Episodic memory and mental intactness	Early maternal death was linked to lower episodic memory scores at baseline, with a decrease of 0.20 points compared to those without this experience. In contrast, better childhood factors such as enhanced paternal education, a non-agricultural job, food security, and good childhood health were associated with better episodic memory. Higher childhood socioeconomic status (SES) predicted higher baseline cognition in both age groups, though it only protected against cognitive decline at baseline in middle-aged adults.
**29**	Thomas et al. (2024) [63]	Early-Life Parental Affection, Social Relationships in Adulthood, and Later-Life Cognitive Function	US	Cohort	Three waves of the Midlife in the United States (MIDUS) study, telephone interviews and self- administered questionnaires	50+	1,983	Descriptive statistics, structural equation modeling (SEM)	Parental Affection in Childhood	Immediate word list recall, delayed word list recall, backward digit span, number series, counting backward speed task, and category fluency	Significant indirect effects of parental affection on better cognitive function through higher levels of social support (both average social support and family social support) in adulthood in the full sample.
**30**	Zhang et al. (2020) [69]	Early-life socioeconomic status, adolescent cognitive ability, and cognition in late midlife: Evidence from the Wisconsin Longitudinal Study	US	Cohort	The Wisconsin Longitudinal Study Wave 1957, 1964, 1975, 1993, 2004, and 2011	65+	5,880	Descriptive statistics, structural equation models	Parent’s education, parent’s occupation, childhood household income, education	Immediate and delayed recall, digit ordering, letter and category fluency, and a subset of the Wechsler Adult Intelligence Scale similarities test.	Childhood SES had a direct effect on cognition in late midlife (β = 0.072, p < 0.01). Mother’s education (CFI = 0.499), father’s education (CFI = 0.719), father’s occupational education (CFI = 0.682), household income (CFI = 453).
**31**	Yuan et al. (2022) [51]	Heterogeneous adverse childhood experiences and cognitive function in an elderly Chinese population: a cohort study	China	Cohort	The China Health and Retirement Longitudinal Study Wave 1,2,3,4 2011-2018	60+	7,222	Descriptive statistics, χ2 test, rank-sum test, binary logistic models	Abuse (physical and emotional), neglect (physical and emotional), household dysfunction (substance abuse, incarceration, mental illness, violence, parental separation or absence), and living surroundings (bullying and community violence)	Mini-Mental State Examination	Child maltreatment was related to a higher risk of cognitive impairment (OR = 1.37, 95% CI: 1.12 to 1.68), compared with low ACEs participants.
**32**	Greenfield et al. (2021) [66]	Life Course Pathways From Childhood Socioeconomic Status to Later-Life Cognition: Evidence From the Wisconsin Longitudinal Study	US	Cohort	The Wisconsin Longitudinal Study Wave 1957, 1964, 1975, 1993, 2004, and 2011	72	3,706	Descriptive statistics, structural equation modeling (SEM), Confirmatory factor analysis	Childhood SES	Verbal fluency, working memory, immediate and delayed word recall test (episodic memory)	Scholastic performance in adolescence and midlife status attainment together fully mediated associations between childhood SES and both memory and language/executive functioning at age 72. Adolescent scholastic performance was directly associated with later-life cognition, as well as indirectly through midlife status attainment
**33**	Voyer et al. (2023) [40]	Linking Adverse Childhood Experiences and Other Risk Factors to Subjective Cognitive Decline in an Aging Population	US	Cross-sectional	The 2019 Behavioral Risk Factor Surveillance System Survey (BRFSS)	45+	17,042	Descriptive statistics, multivariate logistic regression	ACEs included sexual, physical/psychological and environmental ACEs	Survey respondents were asked if they had “experienced confusion or memory loss that is happening more often or is getting worse” in the past 12 months (15). If a participant responded affirmatively, they were identified as having SCD and were then asked a series of 5 additional questions regarding their level of difficulty with day-to-day activities, whether they needed help with these activities, whether they were able to get help when needed, whether SCD interfered with socialization, and whether they had discussed their confusion or memory loss with a clinician	Two or more ACEs also significantly increased the odds of SCD (AOR, 1.69; 95% CI, 1.36–2.10).
**34**	He and Yang (2024) [46]	Longitudinal association of adverse childhood experiences with cognitive function trajectories among middle-aged and older adults- group-based trajectory modeling	China	Cohort	The China Health and Retirement Longitudinal Study Wave 1,2,3,4 2011-2018	45+	1,679	Descriptive statistics, group-based Trajectory Modelling, multinomial unordered logistic models	Child maltreatment, exposure to violence, parent/sibling death or disability, and parental maladjustment	The assessment of episodic memory encompasses both immediate recall and delayed recall. The assessment of mental intactness includes time orientation, overlapping pentagon drawing, and arithmetic	Three cognitive decline subgroups emerged: low-start decline, high- start stability, and mid-start decline. There is no dose-response relationship between cumulative adverse childhood experiences and cognitive function.
**35**	Lee et al. (2021) [67]	Multigenerational Households During Childhood and Trajectories of Cognitive Functioning Among U.S. Older Adults	US	Cohort	The Health and Retirement Study (1998–2014)	51+	8,799	Descriptive statistics, growth curve models	Childhood family structure	Telephone Interview of Cognitive Status	Childhood family structure was significantly linked to cognitive functioning. Individuals from multigenerational households, including those living with a single parent and grandparents, showed higher cognitive functioning compared to those from two-parent households.
**36**	Gutierrez et al. (2024) [57]	My Parent, Myself, or My Child- Whose Education Matters Most for Trajectories of Cognitive Aging in Middle Age	Mexico	Cohort	The Mexican Health and Aging Study (MHAS)	50+	8,822	Descriptive statistics, linear mixed models	Parental education, education	The Cross-Cultural Cognitive Examination	Lower educational levels in both parents (β = − 0.005) and respondents (β = − 0.013) were linked to faster decline in delayed verbal memory scores, but not in immediate verbal memory scores.
**37**	Lian et al. (2024) [41]	No Association Found- Adverse Childhood Experiences and Cognitive Impairment in Older Australian Adults	Australia	Cross-sectional	The Personality and Total Health (PATH) Through Life Project Wave 4	72-79	1,568	Descriptive statistics, multiple logistic regressions	Childhood SES	Dementia diagnosis in Medical record	No significant association between childhood adversity and the presence of cognitive impairment or dementia across all tested models.
**38**	Nilaweera et al. (2022) [42]	The association between adverse childhood events and later-life cognitive function and dementia risk	France	Cross-sectional	The Etude Santé Psychologique Prévalence Risques et Traitement (ESPRIT) 1999-2001	65+	1,562	Descriptive statistics, logistic regression models, Cox proportional	Abuse or maltreatment, death of a parent, prolonged parental hospitalization, serious illness of a parent or child, poverty and financial difficulties, strict upbringing, war or natural disaster, and frequent conflicts at home.	Diagnostic and Statistical Manual of Mental Disorders, Mini-Mental State Examination, Benton’s Visual Retention Test for visual memory, Isaacs Set Test for verbal fluency	Individuals with multiple adverse childhood events had an increased risk of poor psychomotor speed at baseline, with those experiencing 3-4 events having an odds ratio (OR) of 1.39 and those with ≥ 5 events having an OR of 1.52. Worse verbal fluency was observed in individuals with 3-4 ACEs (OR: 1.34). Early-life abuse/maltreatment (OR: 1.47) and poverty/financial difficulties (OR: 1.53) were associated with worse psychomotor speed.
**39**	Schickedanz et al. (2022) [43]	The Association Between Adverse Childhood Experiences and Positive Dementia Screen in American Older Adults	US	Cross-sectional	The 2017 wave of the Panel Study of Income Dynamics (PSID) and the 2014 PSID Childhood Retrospective Circumstances Survey	65+	1,488	Descriptive statistics, binomial logistic analytic model, adjusted ordered logistic regression	Parent mental illness, parent substance abuse, parent intimate partner violence, parental divorce or separation, deceased or absent parent, physical abuse, sexual abuse, emotional abuse, and neglect experienced	The AD8 dementia screen, a judgment, orientation, and function participant or informant-reported, 8-item measure of worsening cognitive impairment used to discriminate dementia from normal cognition.	Older adults with ≥ 4 ACEs had higher rates of a positive dementia screen (AD8 score ≥ 2 points) compared to those with no ACEs (26.6% vs. 16.3%, p = 0.034). Respondents with ≥ 4 ACEs also had higher odds of a 1-point increase in AD8 score across all intervals (adjusted odds ratio: 1.79, 95% CI: 1.05–3.04).
**40**	Hendrie et al. (2018) [58]	The Association of Early Life Factors and Declining Incidence Rates of Dementia in an Elderly Population of African Americans	Nigeria	Cohort	The Indianapolis-Ibadan project 1992-2009	70+	3,276	Descriptive statistics, t-tests, Fisher’s exact tests, Cox proportional hazards regression models	Education	The community screening interview for dementia (CSID)	Significant interaction (p = 0.0477) between education and childhood for AD risk. Higher education level associated with reduced AD risk (HR = 0.87).
**41**	Künzi et al. (2024) [61]	The impact of early adversity on later life health, lifestyle, and cognition	UK	Cohort	The English Longitudinal Study of Ageing (ELSA) and the UK Biobank	40-73	515,013	Descriptive statistics, path analysis, The Full Information Maximum Likelihood (FIML) estimation method	Physical assault, sexual assault, parental abuse, deprivation, physical neglect, sexual abuse, emotional neglect, physical abuse, emotional abuse	Immediate memory recall, visual declarative memory	Parental abuse was associated with poorer immediate memory and verbal fluency. Deprivation at age 10 predicted worse memory and verbal fluency. Physical neglect in childhood was associated with more errors in visual memory tasks.
**42**	Ma et al. (2021) [47]	The influence of childhood adversities on mid to late cognitive function: From the perspective of life course	China	Cohort	The China Health and Retirement Longitudinal Study Wave 2,3 2013-2015	45+	9,942	Descriptive statistics, structural equation models	Parents’ education, father’s occupation, self-assessed household economic status, lack of friends, parental mental health problems, and parent–child relationships	Telephone Interview of Cognitive Status	Parental mental health problems during childhood and poor parent–child relationships both significantly influenced cognitive decline (β = -0.190, SE = 0.011; β = -0.033, SE = 0.012).
**43**	Tsang et al. (2022) [62]	The long arm of childhood socioeconomic deprivation on mid- to later-life cognitive trajectories: A cross-cohort analysis	UK, US	Cohort	The Whitehall II study, the Health and Retirement Study, and the Kame Project	50+	15,309	Descriptive statistics, latent class mixed models, logistic regressions	Parental education, parental unemployment, and family financial hardship	Mini–Mental State Examination, verbal memory word free recall, written naming task of words beginning with the letter “S.”, the Telephone Interview for Cognitive Status	Lower childhood SES was generally associated with a faster cognitive decline trajectory, placing individuals in lower cognitive trajectory classes.
**44**	Lor et al. (2023) [64]	What is the association between adverse childhood experiences and late-life cognitive decline? Study of Healthy Aging in African Americans (STAR) cohort study	US	Cohort	The Study of Healthy Aging in African Americans 2018-2019	50+	764	Descriptive statistics, linear mixed models	Parents’ divorce or separation, a parent remarrying, witnessing domestic violence, substance abuse by a family member, parental job loss, parental incarceration, serious illness of a family member, and the death of a mother or father.	Executive function (constructed from category fluency, phonemic/letter fluency and working memory) and verbal episodic memory	Compared with no ACEs, two ACEs (β=0.117; 95% CI 0.052 to 0.182), three ACEs (β=0.075; 95% CI 0.007 to 0.143) and four or more ACEs (β=0.089; 95% CI 0.002 to 0.158) were associated with less decline in executive function

*aOR: Adjusted Odds Ratio; ACE: Adverse Childhood Experiences; AD8: Alzheimer’s Disease 8 (screening test); β: Beta (coefficient); CI: Confidence Interval; CFI: Comparative Fit Index; DSM-IV: Diagnostic and Statistical Manual of Mental Disorders, 4th Edition; HR: Hazard Ratio; MCI: Mild Cognitive Impairment; MMSE: Mini-Mental State Examination; OR: Odds Ratio; P: P-value; QMCI: Quantitative Measure of Cognitive Impairment; SCD: Subjective Cognitive Decline; SCC: Subjective Cognitive Complaints; SES: Socioeconomic Status; SEP: Socioeconomic Position; SD: Standard Deviation; SDS: Social Dominance Scale; TMTB: Trail Making Test B.

## Results

### Study characteristics

Research on the relationship between childhood health, SES, and later-life dementia spans globally. This review identified a notable focus on resource-rich countries. The US published 15 studies [26–29,32,33,40,43,63–69], plus a multicentre study with the UK [62]. China published eight studies [44–51], and Japan published four studies [30,31,55,56]. Two studies were conducted across Europe [52,53], while two others were conducted in the UK [61], including one in Scotland [59]. The remaining countries–Australia [41], Finland [54], France [42], India [35], Indonesia [37], Ireland [36], Malaysia [39], Mexico [57], Nigeria [58], South Africa [34], Sweden [60], and Vietnam [38]–each have only one study. This highlights a diverse but uneven distribution of research.

Age ranges varied across participants, but the baseline was at least 40 years old because all studies aimed to evaluate any links between childhood health and childhood SES and cognitive function later in life. Sample sizes across studies varied significantly, ranging from 121 [29] to 515,013 [61], with the risk that the smaller population studies may lack sufficient power to detect meaningful differences or associations.

A number of population databases were noted across the 44 studies. Sixteen studies analyzed data from the Health and Retirement Studies International Family of Studies, including the Health and Retirement Study (HRS) [62,67], the China Health and Retirement Longitudinal Study (CHARLS) [45–51], the Indonesia Family Life Survey (IFLS) [37], the English Longitudinal Study of Aging (ELSA) [61], the Survey of Health, Aging, and Retirement in Europe (SHARE) [52,53], the Mexican Health and Aging Study (MHAS) [57], the Irish Longitudinal Study on Ageing (TILDA) [36], and the Longitudinal Ageing Study in India (LASI) [35]. Using these validated population datasets offers several strengths, such as harmonized data collection, diverse cross-cultural samples, and longitudinal assessments of cognitive function. Longitudinal data tracking cognitive changes over time allows for in-depth analyses of the long-term impact of childhood experiences. However, a notable limitation of this methodology is reliance on retrospective self-reports of childhood health and SES and associated recall bias.

Other sources of data across the studies were retrieved from surveys, such as the Behavioral Risk Factor Surveillance System Survey (BRFSS) [27,32,40], the Japan Gerontological Evaluation Study (JAGES) [31,55,56], and the Wisconsin Longitudinal Study (WLS) [66,69]. Each survey drew from prevalidated cognitive assessment tools, such as dementia diagnosis, medical records, and MMSE. These tools are highly reliable in identifying dementia-related outcomes. Papers drawn from survey data had smaller study populations when compared with larger longitudinal datasets. This may reduce the generalizability and cross-cultural applicability of the findings, but they offer contextual, country-specific foci.

In summary, compared to other studies, those in the HRS family excel at capturing long-term, cross-cultural trends, though they still encounter challenges related to recall bias.

A number of pre-validated measurement instruments were applied across the evidence. For cognitive function and dementia diagnosis, most studies utilized the Mini-Mental State Examination (MMSE) [35,36,38,42,44,51,55,56,59]. MMSE is a validated tool for assessing cognitive function in community settings due to its quick administration, ease of use, and robust validation across diverse populations [70]. The MMSE’s 11 items evaluate five domains of cognitive function: orientation, registration, attention, recall, and language, providing a comprehensive overview of an individual’s cognitive status. This makes it particularly suitable for large-scale surveys and effective screening of cognitive impairment in older populations [70]. Other tools included the Cognitive Abilities Screening Instrument (CASI) [68], Community Screening Interview for Dementia (CSI ‘D’) [58], Cross-Cultural Cognitive Examination (CCCE) [57], Delayed Word Recall Test (DWRT) [44,63,65,66], Geriatric Mental State (GMS) [39], ICD-8, 9, & 10 [54], medical records [41,60], Quick Mild Cognitive Impairment (QMCI) [30], Telephone Interview for Cognitive Status (TICS) [37,45,62], Montreal Cognitive Assessment (MoCA-SA) [26], and Wechsler Adult Intelligence Scale (WAIS) [69].

Variables related to childhood health, childhood SES, ACEs, parental engagement, education, nutritional status, living conditions, and books read were included, ensuring a comprehensive evaluation of the link between childhood health, childhood SES, and cognitive impairment or dementia in later life.

Only 11 papers reported response rates [26,30,31,34,36,40,48,51,55–57]. The highest response rate (91.8%) was observed in the MHAS from the HRS family of surveys [57], whereas the lowest response rate (35.3%) was in a Japanese cross-sectional study [30]. This is notable from a methodological perspective, as higher response rates may be subject to response and selection bias. Individuals with better cognitive function, higher SES, or greater engagement with healthcare systems are more likely to participate, therefore overrepresenting the healthier populations. Recognizing these biases is essential when interpreting results, particularly in studies evaluating datasets that measure early-life factors against later-stage cognitive outcomes. Moreover, most studies relied on retrospective information about childhood health and childhood SES, which may be subject to recall bias and/or missing data. To mitigate these biases, a range of advanced statistical techniques such as structural equation modeling (SEM) [63,66], latent growth curve modeling [49], sensitivity analysis [36], and path analysis [61] were used. To determine linear relationships, binary, multinomial, and multivariate regression models were applied [26,28,31,34–37,42,43,55,56,58]. Mixed-effects models evaluated temporal trends [28,50,65]. More advanced regression techniques were employed to assess relative effects. These included Cox proportional hazards models [42,55,56,58], multi-state survival models [53], and group trajectory models [46]. More specific data distributions and between-group comparisons across variables were statistically evaluated using appropriate tests such as chi-square [26,44], ANOVA [44], and Fisher’s exact test [58]. Additionally, quantile regression [38] and marginal structural models [45] were applied to address specific data distributions and explore causal relationships.

### The long arm of childhood circumstances on cognitive function and dementia

A review of all data and information across studies revealed five common themes, presenting factors describing the associations between childhood health and childhood SES, and potential links with dementia and cognitive function. These include: (1) childhood health; (2) childhood educational attainment; (3) family socioeconomic and educational factors; (4) childhood experiences; and (5) childhood reading habits and social interactions.

#### Childhood health.

Several studies identified links between childhood health and cognitive impairment or dementia later in life. Sha et al.’s longitudinal study of 10,533 participants linked better childhood health with 1.1 times higher cognitive performance in middle and older age when compared with those reporting poorer childhood health [49]. Cross-sectional studies report even higher likelihood estimates of 1.4–1.74 across India [35] and the US [26]. Similarly, Kobayashi et al.’s population-based analysis of older people linked poor childhood health history to a 28% reduction in cognitive scores later in life [34]. Donley et al.’s Finnish longitudinal study of 2,682 participants revealed that childhood stress approximately doubled the risk of dementia [54]. Findings related to childhood nutrition were also noteworthy. Momtaz’s (2015) Malaysian cross-sectional study of 2,745 participants revealed that childhood food scarcity nearly doubled the likelihood of cognitive impairment in old age [39]. This finding was slightly higher than those reported in studies conducted in Vietnam [38] and China [48], which linked severe childhood hunger to a 1.5 times increased risk of poor cognitive outcomes. Conversely, analysis of data from the Irish longitudinal study on aging linked improved cognitive functions with childhood infectious diseases. They found the more infectious diseases experienced by children, the better their cognitive function later in life. This study of 2,994 participants posits that each additional infectious disease may be associated with a 0.18-point improvement in cognitive functioning [36].

#### Childhood educational attainment.

Childhood education has also been significantly linked to later-life cognitive function. Koyabishi et al.’s study found that each additional year of education may increase cognitive function scores in later life by 0.09 points [34]. Similarly, Hendrie et al.’s (2018) Nigerian study of 3,276 participants found that each additional year of education reduced the risk of dementia by 7% [58]. These figures are reflected in studies across the US (1.23 times higher risk of dementia with lower education attainment) [68]. In Sweeden, Dekhtyar et al.’s (2015) retrospective analysis of 7,574 students, aggregated to subject-specific performance, found links between lowest grade point average students and a 1.21 times higher risk of dementia in later life [60].

#### Family socioeconomic and educational factors.

This systematic review also indicates a significant body of evidence linking family socioeconomic and education factors with dementia. Studies across India, the US, the UK, and China revealed individuals in midlife or older who had reported experiencing financial hardship during childhood tend to exhibit greater cognitive decline, ranging from 9% to 22% when compared to those with more favorable financial conditions [35,48,49,68]. A slightly higher figure was reported by a cross-study in Indonesia, which found that lack of basic amenities such as electricity, running water, and indoor toilets during childhood was associated with lower cognitive function scores later in life, with a difference of up to 26% [37]. Another cross-sectional study in South Africa found that individuals whose fathers held professional jobs had cognitive scores 0.25 points higher than those whose fathers worked in unskilled manual labor [34]. Additionally, a study conducted in Japan reported that compared to individuals with high SES during childhood, those with low SES and middle SES were 1.29 times and 1.1 times more likely to report subjective memory complaints in later life, respectively [31]. A retrospective study using data from the WLS found that an increase of one standard deviation in childhood SES was associated with memory scores higher by 8% and language/executive function scores higher by 34% at age 72 [66]. Aartsen et al.’s (2019) European longitudinal study of 24,066 participants [52], compared lower childhood SES (disadvantaged) populations with higher SES (advantaged) populations and identified significant reductions in verbal fluency and recall by age 73 in the most disadvantaged groups. A US cross-sectional study [33] of 9,331 participants aged 45-74 linked parental higher educational status with a 0.26 higher average score for their children on cognitive tests in later life. Children of parents who are literate and educated were found to have 0.5 times higher memory scores when compared to lower-educated parents [44].

However, Racine et al.’s Scottish cohort analysis [59] found that a lower education status mother may predict an increased risk of cognitive decline, yet the study indicated additional confounding factors. They found father’s employment as manual labor, or reduced educational attainment, had no impact on cognitive outcome measures.

#### Childhood experiences.

The childhood experiences summarized in this review encompass various factors influencing children’s development. These experiences are categorized into key groups: abuse and neglect, including physical, emotional, and sexual abuse, lack of parental care and attention or absence of peer relationships; family-related issues, such as parental loss, divorce, remarriage, domestic violence, or parental mental health problems and substance abuse; and adverse living environments including unsafe neighborhoods.

There is a wealth of evidence supporting links between high levels of ACEs and increased risk of dementia. Longitudinal studies in Japan [55,56] and the US [32], indicate individuals with three or more ACEs had a 1.78 to 3.25 times higher risk of developing dementia compared to those without such experiences. A French study estimated that individuals with three to four ACEs had a 1.39 times higher risk of reduced psychomotor speed, and risks increased with each additional ACE [42]. Those with five or more ACEs had a 1.52 times higher risk compared to individuals with two or fewer ACEs [42]. Lor et al.’s study of healthy aging in Africa, with 764 participants linked to two ACEs posed an 11.7% higher risk of cognitive decline, those with three ACEs had a 7.5% higher risk, and those with four or more ACEs had an 8.9% higher risk, perhaps indicating ACES is not cumulative [64].

Moving on from comparative studies determining how many ACES cumulatively affect cognitive functioning in later life, we can assert that there is a link. This is supported by a large cross-sectional US study (N = 1,488), which identified a 10.3% higher prevalence of dementia in older adults with four or more ACEs [43].

Childhood sexual abuse is also associated with a 1.37 increased risk of cognitive decline in later life [51]. Another US-based study found this even higher, citing that individuals who experienced sexual abuse as a child had a 2.83 times greater likelihood of subjective cognitive decline when compared to those without [27].

Parent-child relationships are also highly important, with one linking medium to high levels of positive parental involvement during childhood with increased cognitive score outcomes [30]. A large UK longitudinal evaluation of 515,013 participants revealed the significant impact of parental abuse on future cognitive development, memory, verbal fluency, and recall [61]. Other negative family events, (such as parental remarriage, and parent death) have been linked to lower cognitive outcomes in later life. Specifically, parental remarriage was associated with a 0.11-point decrease in cognitive test scores, maternal death with a 0.18-point decrease, and paternal death with a 0.11-point decrease [28]. Conversely, an Australian study of 1568 participants revealed no links between ACEs and diagnoses of dementia [41].

#### Childhood reading habits and social interactions.

There is also evidence supporting childhood reading habits and social interactions and potential links with cognitive function. A Japanese study found regular reading resulted in 3.11 higher test scores in later life when compared to non-readers [30]. Individuals with more books in the household had higher cognitive outcome scores and a 21% risk reduction of dementia [37,53]. Furthermore, longitudinal studies in China found that individuals who faced difficulties in establishing peer relationships during childhood scored 0.208 points lower on cognitive tests and had a 21% higher risk of developing dementia compared to those with positive peer relationships [45,47].

## Discussion

This study highlights poor childhood health, low educational attainment, adverse socioeconomic conditions, ACEs, limited reading habits, and inadequate social interactions increase the risk of dementia later in life. It could be explained that chronic illnesses and malnutrition during childhood disrupt neurodevelopment [71], negatively affecting brain structure and function. Such disruptions result in reduced grey matter volume and alterations in white matter integrity, which are known contributors to long-term cognitive decline [72–74]. However, one study reported that children who experienced multiple infectious diseases during childhood scored higher on cognitive assessments [36]. This counterintuitive finding suggests a need for further research to understand potential mechanisms linking childhood immune challenges and cognitive outcomes.

Several studies showed that higher education levels are consistently associated with better cognitive function. This aligns with prior evidence suggesting that higher education levels were linked to slower brain function decline, lower prevalence of cognitive disorders [75,76], and better short- and long-term memory performance throughout life [77]. Childhood SES also plays a pivotal role in later cognitive outcomes. Children from low-income families face barriers to adequate nutrition, healthcare, educational opportunities, and limited access to intellectually stimulating activities [78–82]. Moreover, substandard living conditions—such as homes lacking electricity or running water—exacerbate childhood stress and impair development [37,83,84]. These factors hinder optimal brain development and predispose children to poorer cognitive function [85]. Furthermore, a stable family structure and higher parental education levels significantly contribute to cognitive function later in life [86]. This can be explained by the fact that parents with higher education levels, leading to stable and well-paying jobs, are better positioned to provide resources, opportunities, academic support, and an environment conducive to cognitive development [87–89].

ACEs, including abuse, neglect, and family dysfunction, have consistently been associated with poorer cognitive outcomes. These findings corroborate previous evidence suggesting that stress and trauma during critical periods of brain development lead to structural and functional changes in the brain [90–92]. Such alterations often affect regions involved in stress regulation and cognitive functioning, resulting in long-term impairments. Additionally, the presence of both parents in a family, compared to single-parent families or those without parental presence, has been linked to better emotional regulation and social skills, which are crucial for cognitive health [93]. However, one cross-sectional study conducted in Australia did not find an association between ACEs and cognitive impairment [41]. This finding may be explained by the fact that the study only included participants from Canberra (the capital) and Queanbeyan, which are both relatively affluent areas [94]. These cities have a higher concentration of wealth, and individuals residing in such environments may have experienced lower levels of childhood adversity compared to populations from less advantaged regions. As a result, this could have influenced the study’s findings.

A notable finding in this study is that access to books during childhood is associated with a reduced risk of dementia. While evidence on this topic is limited in previous research, this finding can be explained by the cognitive stimulation provided by reading, which enhances language skills and promotes lifelong learning—factors crucial for maintaining cognitive resilience [95]. Children engaged in reading activities are more likely to develop critical thinking and comprehension skills, contributing to improved educational and cognitive outcomes [95]. However, as previously noted, limited research has explored the influence of childhood reading on cognitive function in later life, emphasizing the need for further studies to provide a clearer understanding of its role in cognitive development.

Finally, the importance of social interactions during childhood was also recorded. This finding can be explained by the role of social interactions in developing key skills such as communication, emotional regulation, and problem-solving [96]. Children who experience social isolation are deprived of opportunities to cultivate these skills, which can adversely impact cognitive health [97].

This study synthesizes global evidence, emphasizing the importance of early-life interventions in preventing cognitive decline. Public health initiatives should prioritize enhancing children’s physical and mental health while addressing socioeconomic inequalities. These strategies not only benefit individuals but also alleviate the societal and economic burden of cognitive decline and dementia, aligning with WHO guidelines [4]. Beyond the scope of these guidelines, this study shows that promoting reading habits from an early age may serve as a potential protective factor for cognitive health, supporting existing theories on cognitive reserve. Future research is needed to clarify the causal relationship between reading habits and cognitive development. Additionally, a deeper exploration of the impact of childhood infectious diseases on later-life cognition is warranted to inform future preventive strategies.

This study may have some limitations. Firstly, it was restricted to English-language papers published between 2014 and 2024, potentially excluding relevant research published in other languages or from earlier periods. Secondly, a substantial proportion of the studies were cross-sectional, which limits the ability to establish causality between risk factors and dementia. Thirdly, the variation in sample sizes among the included studies could affect the reliability of the results. There were also inconsistencies in the criteria used to define exposure factors in some studies may impact the comparability and validity of the findings and, as many studies drew from the same datasets, (such as CHARLS, WLS, and JAGES), and thus, results may be skewed through duplicate counting.

## Conclusions and implications

Childhood health, childhood educational attainment, family socioeconomic and educational factors, childhood experiences, and childhood reading habits and social interactions were all linked to the risk of cognitive impairment and dementia in later life. To establish causal links between early-life risk factors and later cognitive impairment, further superior longitudinal studies are essential. Policymakers should prioritize early childhood development programs that combine health, nutrition, education, and social support to mitigate the incidence of dementia and cognitive impairment in later life. Over 60% of studies included in this review concentrated in a few wealthy nations (the US, China, and Japan), likely reflecting disparities in research capacity and funding priorities at the global level. It also raises concerns about whether the findings, which specific socioeconomic and cultural contexts may influence, can be generalized to other parts of the world, particularly those with fewer resources and different social structures. Future research should prioritize broader geographic inclusion to ensure a more comprehensive and equitable understanding of how childhood experiences influence dementia risk.

## Supporting information

S1 Appendix PRISMA checklist. (DOCX)

S2 Appendix Systematic review screening process. (XLSX)

## References

[1] WHO. Ageing and health 2024. 2024. Available from: https://www.who.int/news-room/fact-sheets/detail/ageing-and-health#:~:text=By%202050%2C%20the%20world’s%20population,2050%20to%20reach%20426%20million

[2] James SL, Abate D, Abate KH, Abay SM, Abbafati C, Abbasi N, et al.; GBD 2017 Disease and Injury Incidence and Prevalence Collaborators. Global, regional, and national incidence, prevalence, and years lived with disability for 354 diseases and injuries for 195 countries and territories, 1990-2017: a systematic analysis for the Global Burden of Disease Study 2017. Lancet. 2018;392(10159):1789–858. doi: 10.1016/S0140-6736(18)32279-7 30496104 PMC6227754

[3] Livingston G, Huntley J, Sommerlad A, Ames D, Ballard C, Banerjee S, et al. Dementia prevention, intervention, and care: 2020 report of the Lancet Commission. Lancet. 2020;396(10248):413–46. doi: 10.1016/S0140-6736(20)30367-6 32738937 PMC7392084

[4] World Health Organization. Risk reduction of cognitive decline and dementia: WHO guidelines. Geneva: World Health Organization; 2019. 31219687

[5] Neufeld LM, Beal T, Larson LM, Cattaneo FD. Global Landscape of Malnutrition in Infants and Young Children. Nestle Nutr Inst Workshop Ser. 2020;93:1–14. doi: 10.1159/000503315 31991423

[6] Group WB, UNICEF. 1 in 6 children lives in extreme poverty, World Bank-UNICEF analysis shows 2020. Available from: https://www.unicef.org.uk/press-releases/1-in-6-children-lives-in-extreme-poverty-world-bank-unicef-analysis-shows/#:~:text=The%20pre%2DCOVID%2D19%20analysis,them%20in%20sub%2DSaharan%20Africa

[7] Korhonen K, Leinonen T, Tarkiainen L, Einiö E, Martikainen P. Childhood socio-economic circumstances and dementia: prospective register-based cohort study of adulthood socio-economic and cardiovascular health mediators. Int J Epidemiol. 2023;52(2):523–35. doi: 10.1093/ije/dyac205 36343014 PMC10114069

[8] Zhang Z, Gu D, Hayward MD. Childhood nutritional deprivation and cognitive impairment among older Chinese people. Soc Sci Med. 2010;71(5):941–9. doi: 10.1016/j.socscimed.2010.05.013 20591545 PMC4476240

[9] Hernandez-Ruiz V, Letenneur L, Fülöp T, Helmer C, Roubaud-Baudron C, Avila-Funes J-A, et al. Infectious diseases and cognition: do we have to worry?. Neurol Sci. 2022;43(11):6215–24. doi: 10.1007/s10072-022-06280-9 35867217 PMC9305033

[10] Pandya D, Johnson TP. Chronic and delayed neurological manifestations of persistent infections. Curr Opin Neurol. 2023;36(3):198–206. doi: 10.1097/WCO.0000000000001154 37078647 PMC10155704

[11] Yahirun JJ, Vasireddy S, Hayward MD. The Education of Multiple Family Members and the Life-Course Pathways to Cognitive Impairment. J Gerontol B Psychol Sci Soc Sci. 2020;75(7):e113–28. doi: 10.1093/geronb/gbaa039 32215643 PMC7424275

[12] Zajacova A, Lawrence EM. The Relationship Between Education and Health: Reducing Disparities Through a Contextual Approach. Annu Rev Public Health. 2018;39 273–89. doi: 10.1146/annurev-publhealth-031816-044628 29328865 PMC5880718

[13] Luo Y, Waite LJ. The impact of childhood and adult SES on physical, mental, and cognitive well-being in later life. J Gerontol B Psychol Sci Soc Sci. 2005;60(2):S93–101. doi: 10.1093/geronb/60.2.s93 15746030 PMC2505177

[14] McEniry M. Early-life conditions and older adult health in low- and middle-income countries: a review. J Dev Orig Health Dis. 2013;4(1):10–29. doi: 10.1017/S2040174412000499 23316272 PMC3540412

[15] Krugers HJ, Arp JM, Xiong H, Kanatsou S, Lesuis SL, Korosi A, et al. Early life adversity: Lasting consequences for emotional learning. Neurobiol Stress. 2016;6:14–21. doi: 10.1016/j.ynstr.2016.11.005 28229105 PMC5314442

[16] Cooper K, Stewart K. Does Household Income Affect children’s Outcomes? A Systematic Review of the Evidence. Child Ind Res. 2020;14(3):981–1005. doi: 10.1007/s12187-020-09782-0

[17] Wang X-J, Xu W, Li J-Q, Cao X-P, Tan L, Yu J-T. Early-Life Risk Factors for Dementia and Cognitive Impairment in Later Life: A Systematic Review and Meta-Analysis. J Alzheimers Dis. 2019;67(1):221–9. doi: 10.3233/JAD-180856 30636739

[18] Chase E, Statham J. Childhood Wellbeing: A Brief Overview. London Childhood Wellbeing Research Centre, 2010.

[19] Suglia SF, Saelee R, Guzmán IA, Elsenburg LK, Clark CJ, Link BG, et al. Child socioeconomic status, childhood adversity and adult socioeconomic status in a nationally representative sample of young adults. SSM Popul Health. 2022;18:101094. doi: 10.1016/j.ssmph.2022.101094 35601218 PMC9118918

[20] Page MJ, McKenzie JE, Bossuyt PM, Boutron I, Hoffmann TC, Mulrow CD, et al. The PRISMA 2020 statement: an updated guideline for reporting systematic reviews. BMJ. 2021;372:n71. doi: 10.1136/bmj.n71 33782057 PMC8005924

[21] Amir-Behghadami M, Janati A. Population, Intervention, Comparison, Outcomes and Study (PICOS) design as a framework to formulate eligibility criteria in systematic reviews. Emerg Med J. 2020;37(6):387. doi: 10.1136/emermed-2020-209567 32253195

[22] Brown P, Brunnhuber K, Chalkidou K, Chalmers I, Clarke M, Fenton M, et al. How to formulate research recommendations. BMJ. 2006;333(7572):804–6. doi: 10.1136/bmj.38987.492014.94 17038740 PMC1602035

[23] Higgins J, Thomas J, Chandler J, Cumpston M, Li T, Page M, et al. Cochrane handbook for systematic reviews of interventions. John Wiley & Sons; 2019. 10.1002/14651858.ED000142 PMC10284251 31643080

[24] Hannes K, Lockwood C, Pearson A. A comparative analysis of three online appraisal instruments’ ability to assess validity in qualitative research. Qual Health Res. 2010;20(12):1736–43. doi: 10.1177/1049732310378656 20671302

[25] Munn Z, Barker TH, Moola S, Tufanaru C, Stern C, McArthur A, et al. Methodological quality of case series studies: an introduction to the JBI critical appraisal tool. JBI Evid Synth. 2020;18(10):2127–33. doi: 10.11124/JBISRIR-D-19-00099 33038125

[26] Lee VM, Hargrave AS, Lisha NE, Huang AJ. Adverse Childhood Experiences and Aging-Associated Functional Impairment in a National Sample of Older Community-Dwelling Adults. J Gen Intern Med. 2023;38(15):3362–71. doi: 10.1007/s11606-023-08252-x 37532875 PMC10682434

[27] Brown MJ, Kaur A, James T, Avalos C, Addo PNO, Crouch E, et al. Adverse Childhood Experiences and Subjective Cognitive Decline in the US. J Appl Gerontol. 2022;41(4):1090–100. doi: 10.1177/07334648211058718 34898321 PMC8969160

[28] Gold AL, Meza E, Ackley SF, Mungas DM, Whitmer RA, Mayeda ER, et al. Are adverse childhood experiences associated with late-life cognitive performance across racial/ethnic groups: results from the Kaiser Healthy Aging and Diverse Life Experiences study baseline. BMJ Open. 2021;11(2):e042125. doi: 10.1136/bmjopen-2020-042125 33550246 PMC7925876

[29] Halpin AB, MacAulay RK, Boeve AR, D’Errico LM, Michaud S. Are Adverse Childhood Experiences Associated with Worse Cognitive Function in Older Adults? J Int Neuropsychol Soc. 2022;28(10):1029–38. doi: 10.1017/S1355617721001272 34794526

[30] Morita A, Fujiwara T. Association between childhood parental involvement and late-life cognitive function: A population-based cross-sectional study among cognitively intact community-dwelling older adults in Japan. Geriatr Gerontol Int. 2021;21(9):794–801. doi: 10.1111/ggi.14238 34318571

[31] Nishizawa T, Morita A, Fujiwara T, Kondo K. Association between childhood socioeconomic status and subjective memory complaints among older adults: results from the Japan Gerontological Evaluation Study 2010. Int Psychogeriatr. 2019;31(12):1699–707. doi: 10.1017/S1041610219000814 31317850

[32] Baiden P, Cassidy J, Panisch LS, LaBrenz CA, Onyeaka HK. Association of adverse childhood experiences with subjective cognitive decline in adulthood: Findings from a population-based study. Aging Ment Health. 2022;26(11):2214–22. doi: 10.1080/13607863.2021.2017848 34957876

[33] Filigrana P, Moon J-Y, Gallo LC, Fernández-Rhodes L, Perreira KM, Daviglus ML, et al. Childhood and Life-Course Socioeconomic Position and Cognitive Function in the Adult Population of the Hispanic Community Health Study/Study of Latinos. Am J Epidemiol. 2023;192(12):2006–17. doi: 10.1093/aje/kwad157 37420108 PMC10988221

[34] Kobayashi LC, Glymour MM, Kahn K, Payne CF, Wagner RG, Montana L, et al. Childhood deprivation and later-life cognitive function in a population-based study of older rural South Africans. Soc Sci Med. 2017;190:20–8. doi: 10.1016/j.socscimed.2017.08.009 28837862 PMC5915343

[35] Muhammad T, Debnath P, Srivastava S, Sekher TV. Childhood deprivations predict late-life cognitive impairment among older adults in India. Sci Rep. 2022;12(1):12786. doi: 10.1038/s41598-022-16652-y 35896620 PMC9329336

[36] Rotstein A, Levine SZ. Childhood infectious diseases and old age cognitive functioning: a nationally representative sample of community-dwelling older adults. Int Psychogeriatr. 2021;33(1):75–82. doi: 10.1017/S1041610220001404 32703324 PMC8482372

[37] Maharani A. Childhood Socioeconomic Status and Cognitive Function Later in Life: Evidence From a National Survey in Indonesia. J Geriatr Psychiatry Neurol. 2020;33(4):214–22. doi: 10.1177/0891988719874120 31505999

[38] Korinek K, Zimmer Z, Teerawichitchainan B, Young Y, Cao Manh L, Toan TK. Cognitive function following early life war-time stress exposure in a cohort of Vietnamese older adults. Soc Sci Med. 2024;349:116800. doi: 10.1016/j.socscimed.2024.116800 38640743 PMC11105098

[39] Momtaz YA, Haron SA, Hamid TA, Ibrahim R, Masud J. Does food insufficiency in childhood contribute to dementia in later life? Clin Interv Aging. 2014;10:49–53. doi: 10.2147/CIA.S69220 25565786 PMC4279670

[40] Voyer H, Aytur S, Tanda N. Linking Adverse Childhood Experiences and Other Risk Factors to Subjective Cognitive Decline in an Aging Population. Prev Chronic Dis. 2023;20:E115. doi: 10.5888/pcd20.230182 38127683 PMC10756650

[41] Lian J, Kiely KM, Callaghan BL, Eramudugolla R, Mortby M, Anstey KJ. No Association Found: Adverse Childhood Experiences and Cognitive Impairment in Older Australian Adults. J Prev Alzheimers Dis. 2024;11(6):1818–25. doi: 10.14283/jpad.2024.133 39559893 PMC11573808

[42] Nilaweera D, Freak-Poli R, Gurvich C, Ritchie K, Chaudieu I, Ancelin M-L, et al. The association between adverse childhood events and later-life cognitive function and dementia risk. J Affect Disord. 2022;304:128–32. doi: 10.1016/j.jad.2022.02.062 35219735

[43] Schickedanz HB, Jennings LA, Schickedanz A. The Association Between Adverse Childhood Experiences and Positive Dementia Screen in American Older Adults. J Gen Intern Med. 2022;37(10):2398–404. doi: 10.1007/s11606-021-07192-8 34782990 PMC9360371

[44] Chai ZH, Jiang CQ, Jin YL, Zhu F, Cheng KK, Lam TH, et al. Association between childhood parental literacy and late-life cognitive function: The Guangzhou Biobank Cohort Study. Public Health. 2024;237:354–60. doi: 10.1016/j.puhe.2024.10.038 39515220

[45] Zhou Z, Zhao H. Childhood Peer Relationships and Dementia Risk in Chinese Older Adults: A Mediation Analysis. Int J Geriatr Psychiatry. 2024;39(11):e70022. doi: 10.1002/gps.70022 39558464

[46] He X, Yang H. Longitudinal association of adverse childhood experiences with cognitive function trajectories among middle-aged and older adults: group-based trajectory modeling. Front Psychiatry. 2024;15:1440265. doi: 10.3389/fpsyt.2024.1440265 39165500 PMC11333235

[47] Ma J, Yang Y, Wan Y, Shen C, Qiu P. The influence of childhood adversities on mid to late cognitive function: From the perspective of life course. PLoS One. 2021;16(8):e0256297. doi: 10.1371/journal.pone.0256297 34398901 PMC8366991

[48] Yang L, Wang Z. Early-Life Conditions and Cognitive Function in Middle-and Old-Aged Chinese Adults: A Longitudinal Study. Int J Environ Res Public Health. 2020;17(10):3451. doi: 10.3390/ijerph17103451 32429157 PMC7277849

[49] Sha T, Yan Y, Cheng W. Associations of childhood socioeconomic status with mid-life and late-life cognition in Chinese middle-aged and older population based on a 5-year period cohort study. Int J Geriatr Psychiatry. 2018;33(10):1335–45. doi: 10.1002/gps.4930 29971839 PMC6174994

[50] Lin L, Cao B, Chen W, Li J, Zhang Y, Guo VY. Association of Adverse Childhood Experiences and Social Isolation With Later-Life Cognitive Function Among Adults in China. JAMA Netw Open. 2022;5(11):e2241714. doi: 10.1001/jamanetworkopen.2022.41714 36367722 PMC9652754

[51] Yuan M, Qin F, Xu C, Fang Y. Heterogeneous adverse childhood experiences and cognitive function in an elderly Chinese population: a cohort study. BMJ Open. 2022;12(6):e060477. doi: 10.1136/bmjopen-2021-060477 35688592 PMC9189840

[52] Aartsen MJ, Cheval B, Sieber S, Van der Linden BW, Gabriel R, Courvoisier DS, et al. Advantaged socioeconomic conditions in childhood are associated with higher cognitive functioning but stronger cognitive decline in older age. Proc Natl Acad Sci U S A. 2019;116(12):5478–86. doi: 10.1073/pnas.1807679116 30804194 PMC6431198

[53] Lewis NA, Yoneda T, Melis RJF, Mroczek DK, Hofer SM, Muniz-Terrera G. Availability of Cognitive Resources in Early Life Predicts Transitions Between Cognitive States in Middle and Older Adults From Europe. Innov Aging. 2023;7(9):igad124. doi: 10.1093/geroni/igad124 38034934 PMC10682867

[54] Donley GAR, Lönnroos E, Tuomainen T-P, Kauhanen J. Association of childhood stress with late-life dementia and Alzheimer’s disease: the KIHD study. Eur J Public Health. 2018;28(6):1069–73. doi: 10.1093/eurpub/cky134 30020441

[55] Tani Y, Fujiwara T, Kondo K. Adverse Childhood Experiences and Dementia: Interactions With Social Capital in the Japan Gerontological Evaluation Study Cohort. Am J Prev Med. 2021;61(2):225–34. doi: 10.1016/j.amepre.2021.01.045 33985835

[56] Tani Y, Fujiwara T, Kondo K. Association Between Adverse Childhood Experiences and Dementia in Older Japanese Adults. JAMA Netw Open. 2020;3(2):e1920740. doi: 10.1001/jamanetworkopen.2019.20740 32031646

[57] Gutierrez S, Meza E, Glymour MM, Torres JM. My Parent, Myself, or My Child: Whose Education Matters Most for Trajectories of Cognitive Aging in Middle Age? Am J Epidemiol. 2024;193(5):695–706. doi: 10.1093/aje/kwad108 37116072 PMC11484617

[58] Hendrie HC, Smith-Gamble V, Lane KA, Purnell C, Clark DO, Gao S. The Association of Early Life Factors and Declining Incidence Rates of Dementia in an Elderly Population of African Americans. J Gerontol B Psychol Sci Soc Sci. 2018;73(suppl_1):S82–9. doi: 10.1093/geronb/gbx143 29669098 PMC6018682

[59] Racine Maurice S, Hébert A, Turcotte V, Potvin O, Hudon C, Duchesne S. Childhood Socioeconomic Status Does Not Predict Late-Life Cognitive Decline in the 1936 Lothian Birth Cohort. Front Psychol. 2021;12:679044. doi: 10.3389/fpsyg.2021.679044 34248779 PMC8265392

[60] Dekhtyar S, Wang H-X, Scott K, Goodman A, Koupil I, Herlitz A. A Life-Course Study of Cognitive Reserve in Dementia--From Childhood to Old Age. Am J Geriatr Psychiatry. 2015;23(9):885–96. doi: 10.1016/j.jagp.2015.02.002 25746486

[61] Künzi M, Gheorghe DA, Gallacher J, Bauermeister S. The impact of early adversity on later life health, lifestyle, and cognition. BMC Public Health. 2024;24(1):3294. doi: 10.1186/s12889-024-20768-3 39604955 PMC11600748

[62] Tsang RSM, Gallacher JE, Bauermeister S. The long arm of childhood socioeconomic deprivation on mid- to later-life cognitive trajectories: A cross-cohort analysis. Alzheimers Dement (Amst). 2022;14(1):e12322. doi: 10.1002/dad2.12322 35664888 PMC9159813

[63] Thomas PA, Teas E, Friedman E, Barnes LL, Sauerteig-Rolston MR, Ferraro KF. Early-Life Parental Affection, Social Relationships in Adulthood, and Later-Life Cognitive Function. J Aging Health. 2024:8982643241303589. doi: 10.1177/08982643241303589 39629987 PMC12134153

[64] Lor Y, George KM, Gilsanz P, Meunier CC, Peterson RL, Hayes-Larson E, et al. What is the association between adverse childhood experiences and late-life cognitive decline? Study of Healthy Aging in African Americans (STAR) cohort study. BMJ Open. 2023;13(11):e072961. doi: 10.1136/bmjopen-2023-072961 37918928 PMC10626853

[65] Kucharska-Newton AM, Pike JR, Chen J, Coresh J, Sharret AR, Mosley T, et al. Association of Childhood and Midlife Neighborhood Socioeconomic Position With Cognitive Decline. JAMA Netw Open. 2023;6(8):e2327421. doi: 10.1001/jamanetworkopen.2023.27421 37540511 PMC10403777

[66] Greenfield EA, Moorman S, Rieger A. Life Course Pathways From Childhood Socioeconomic Status to Later-Life Cognition: Evidence From the Wisconsin Longitudinal Study. J Gerontol B Psychol Sci Soc Sci. 2021;76(6):1206–17. doi: 10.1093/geronb/gbaa062 32369603 PMC8200350

[67] Lee H, Ryan LH, Ofstedal MB, Smith J. Multigenerational Households During Childhood and Trajectories of Cognitive Functioning Among U.S. Older Adults. J Gerontol B Psychol Sci Soc Sci. 2021;76(6):1161–72. doi: 10.1093/geronb/gbaa165 32951054 PMC8200351

[68] Tom SE, Phadke M, Hubbard RA, Crane PK, Stern Y, Larson EB. Association of Demographic and Early-Life Socioeconomic Factors by Birth Cohort With Dementia Incidence Among US Adults Born Between 1893 and 1949. JAMA Netw Open. 2020;3(7):e2011094. doi: 10.1001/jamanetworkopen.2020.11094 32716513 PMC8794045

[69] Zhang Z, Liu H, Choi S-W. Early-life socioeconomic status, adolescent cognitive ability, and cognition in late midlife: Evidence from the Wisconsin Longitudinal Study. Soc Sci Med. 2020;244:112575. doi: 10.1016/j.socscimed.2019.112575 31606188 PMC6926157

[70] Kurlowicz L, Wallace M. The Mini-Mental State Examination (MMSE). J Gerontol Nurs. 1999;25(5):8–9. doi: 10.3928/0098-9134-19990501-08 10578759

[71] Kirolos A, Goyheneix M, Kalmus Eliasz M, Chisala M, Lissauer S, Gladstone M, et al. Neurodevelopmental, cognitive, behavioural and mental health impairments following childhood malnutrition: a systematic review. BMJ Glob Health. 2022;7(7):e009330. doi: 10.1136/bmjgh-2022-009330 35793839 PMC9260807

[72] Li W-X, Yuan J, Han F, Zhou L-X, Ni J, Yao M, et al. White matter and gray matter changes related to cognition in community populations. Front Aging Neurosci. 2023;15:1065245. doi: 10.3389/fnagi.2023.1065245 36967830 PMC10036909

[73] Cocchi L, Walterfang M, Testa R, Wood SJ, Seal ML, Suckling J, et al. Grey and white matter abnormalities are associated with impaired spatial working memory ability in first-episode schizophrenia. Schizophr Res. 2009;115(2–3):163–72. doi: 10.1016/j.schres.2009.09.009 19837566

[74] Luby J, Belden A, Botteron K, Marrus N, Harms MP, Babb C, et al. The effects of poverty on childhood brain development: the mediating effect of caregiving and stressful life events. JAMA Pediatr. 2013;167(12):1135–42. doi: 10.1001/jamapediatrics.2013.3139 24165922 PMC4001721

[75] Mungas D, Gavett B, Fletcher E, Farias ST, DeCarli C, Reed B. Education amplifies brain atrophy effect on cognitive decline: implications for cognitive reserve. Neurobiol Aging. 2018;68:142–50. doi: 10.1016/j.neurobiolaging.2018.04.002 29798764 PMC5993638

[76] Wilson RS, Yu L, Lamar M, Schneider JA, Boyle PA, Bennett DA. Education and cognitive reserve in old age. Neurology. 2019;92(10):e1041–50. doi: 10.1212/WNL.0000000000007036 30728309 PMC6442015

[77] Lövdén M, Fratiglioni L, Glymour MM, Lindenberger U, Tucker-Drob EM. Education and Cognitive Functioning Across the Life Span. Psychol Sci Public Interest. 2020;21(1):6–41. doi: 10.1177/1529100620920576 32772803 PMC7425377

[78] Christensen DL, Schieve LA, Devine O, Drews-Botsch C. Socioeconomic status, child enrichment factors, and cognitive performance among preschool-age children: results from the Follow-Up of Growth and Development Experiences study. Res Dev Disabil. 2014;35(7):1789–801. doi: 10.1016/j.ridd.2014.02.003 24679548 PMC4997613

[79] Noble KG, Engelhardt LE, Brito NH, Mack LJ, Nail EJ, Angal J, et al. Socioeconomic disparities in neurocognitive development in the first two years of life. Dev Psychobiol. 2015;57(5):535–51. doi: 10.1002/dev.21303 25828052 PMC4821066

[80] Cusick SE, Georgieff MK. The Role of Nutrition in Brain Development: The Golden Opportunity of the “First 1000 Days”. J Pediatr. 2016;175:16–21. doi: 10.1016/j.jpeds.2016.05.013 ; PMCID: PMC4981537 27266965 PMC4981537

[81] Hackman DA, Farah MJ, Meaney MJ. Socioeconomic status and the brain: mechanistic insights from human and animal research. Nat Rev Neurosci. 2010;11(9):651–9. doi: 10.1038/nrn2897 20725096 PMC2950073

[82] Rakesh D, Whittle S, Sheridan MA, McLaughlin KA. Childhood socioeconomic status and the pace of structural neurodevelopment: accelerated, delayed, or simply different? Trends Cogn Sci. 2023;27(9):833–51. doi: 10.1016/j.tics.2023.03.011 37179140 PMC10524122

[83] Evans GW, Schamberg MA. Childhood poverty, chronic stress, and adult working memory. Proc Natl Acad Sci U S A. 2009;106(16):6545–9. doi: 10.1073/pnas.0811910106 19332779 PMC2662958

[84] Fowler PJ, McGrath LM, Henry DB, Schoeny M, Chavira D, Taylor JJ, et al. Housing mobility and cognitive development: Change in verbal and nonverbal abilities. Child Abuse Negl. 2015;48:104–18. doi: 10.1016/j.chiabu.2015.06.002 26184055 PMC4593721

[85] Blair C, Raver CC. Poverty, Stress, and Brain Development: New Directions for Prevention and Intervention. Acad Pediatr. 2016;16(3 Suppl):S30-6. doi: 10.1016/j.acap.2016.01.010 27044699 PMC5765853

[86] Conger RD, Conger KJ, Martin MJ. Socioeconomic Status, Family Processes, and Individual Development. J Marriage Fam. 2010;72(3):685–704. doi: 10.1111/j.1741-3737.2010.00725.x 20676350 PMC2910915

[87] Kantova K. Parental involvement and education outcomes of their children. Applied Economics. 2024;56(48):5683–98. doi: 10.1080/00036846.2024.2314569

[88] Roubinov DS, Boyce WT. Parenting and SES: relative values or enduring principles? Curr Opin Psychol. 2017;15:162–7. doi: 10.1016/j.copsyc.2017.03.001 28503655 PMC5423399

[89] Dubow EF, Boxer P, Huesmann LR. Long-term Effects of Parents’ Education on Children’s Educational and Occupational Success: Mediation by Family Interactions, Child Aggression, and Teenage Aspirations. Merrill Palmer Q (Wayne State Univ Press). 2009;55(3):224–49. doi: 10.1353/mpq.0.0030 20390050 PMC2853053

[90] Anda RF, Felitti VJ, Bremner JD, Walker JD, Whitfield C, Perry BD, et al. The enduring effects of abuse and related adverse experiences in childhood. A convergence of evidence from neurobiology and epidemiology. Eur Arch Psychiatry Clin Neurosci. 2006;256(3):174–86. doi: 10.1007/s00406-005-0624-4 16311898 PMC3232061

[91] Cimeša M, Sokić A, Cimeša B. Childhood Trauma and its Effect on Brain Development: Neurobiological Mechanisms and Implications. 2023.

[92] Smith KE, Pollak SD. Early life stress and development: potential mechanisms for adverse outcomes. J Neurodev Disord. 2020;12(1):34. doi: 10.1186/s11689-020-09337-y 33327939 PMC7745388

[93] Morris AS, Silk JS, Steinberg L, Myers SS, Robinson LR. The Role of the Family Context in the Development of Emotion Regulation. Soc Dev. 2007;16(2):361–88. doi: 10.1111/j.1467-9507.2007.00389.x 19756175 PMC2743505

[94] ABS. Socio-Economic Indexes for Areas (SEIFA), Australia 2021. Available from: https://www.abs.gov.au/statistics/people/people-and-communities/socio-economic-indexes-areas-seifa-australia/latest-release

[95] Chang Y-H, Wu I-C, Hsiung CA. Reading activity prevents long-term decline in cognitive function in older people: evidence from a 14-year longitudinal study. Int Psychogeriatr. 2021;33(1):63–74. doi: 10.1017/S1041610220000812 32498728 PMC8482376

[96] Huang X, Lajoie SP. Social emotional interaction in collaborative learning: Why it matters and how can we measure it? Social Sciences & Humanities Open. 2023;7(1):100447. doi: 10.1016/j.ssaho.2023.100447

[97] Thompson KN, Odgers CL, Bryan BT, Danese A, Milne BJ, Strange L, et al. Trajectories of childhood social isolation in a nationally representative cohort: Associations with antecedents and early adulthood outcomes. JCPP Adv. 2022;2(2):e12073. doi: 10.1002/jcv2.12073 37431453 PMC10242821

