# Supplementary material for: Cognitive impairment and dementia—Are they linked to childhood health and socioeconomic status? A systematic review
Source: PLoS One. 2025 Mar 27;20(3):e0311074. doi: 10.1371/journal.pone.0311074 (PMC11949372; doi:10.1371/journal.pone.0311074)
Supplement: Attachment — Submitted filename: Response to Reviewers.docx [file pone.0311074.s004.docx]

Manchester, 20^th^ January 2025

To: Dr Jordi Gumà, Academic Editor, PLOS ONE

Re: Revised Manuscript (PONE-D-24-40492)

Dear Dr Gumà,

We thank you for your interest in our submission to the PLOS ONE journal and the opportunity to revise and resubmit our manuscript. We appreciate the extensive comments provided following the peer review, each of which we have now considered carefully. In response, we have undertaken a substantial revision of our manuscript in line with the issues and suggestions raised by you and the three reviewers. For clarification, we provide a point-by-point response to these comments below.

We sincerely hope our manuscript now meets your approval for publication in PLOS ONE.

On behalf of the Authors,

Yours sincerely,

Tung Le

**EDITOR COMMENTS**

**Comments from Editor**

Thank you for submitting your manuscript to PLOS ONE. After careful consideration, we feel that it has merit but does not fully meet PLOS ONE’s publication criteria as it currently stands. Therefore, we invite you to submit a revised version of the manuscript that addresses the points raised during the review process.

As the reviewers point out, the manuscript in its current version should be better contextualized, especially by including the most recent publications on the subject. This aspect would substantially improve the relevance of the contribution of your research.

**Authors’ response**
 
Thank you for highlighting this important comment from the reviewers. In response, we have revised the manuscript by incorporating the latest research on the topic. We re-conducted our literature search on December 17, 2024, to ensure we included the most recent publications. Additionally, based on the reviewers' recommendations, we expanded the search terms related to childhood socioeconomic status (SES) to include median household income, parental education level, and parental occupational status to identify more relevant articles for this systematic review.

**Comments from Editor**

In addition, some reviewers indicate in their comments to me the need to improve the writing style of your paper. Therefore, I strongly recommend you to have a professional English style review by a native speaker.

**Authors’ response**

We gratefully acknowledge the suggestions to improve the writing style of our manuscript. In response, we have carefully reviewed and refined the text to ensure clarity, coherence, and polished language. Additionally, the final manuscript was thoroughly reviewed by Dr James Gilleen and Dr Amanda Lee, native English speakers in our research group, to enhance its readability and quality further.

**JOURNAL REQUIREMENTS**

**Journal Requirements**

When submitting your revision, we need you to address these additional requirements.

1. Please ensure that your manuscript meets PLOS ONE's style requirements, including those for file naming. The PLOS ONE style templates can be found at

https://journals.plos.org/plosone/s/file?id=wjVg/PLOSOne_formatting_sample_main_body.pdf and https://journals.plos.org/plosone/s/file?id=ba62/PLOSOne_formatting_sample_title_authors_affiliations.pdf

**Authors’ response**

We have ensured that our manuscript complies with the journal's formatting guidelines, including file naming conventions. The manuscript has been formatted by the PLOS ONE style templates provided at the links, ensuring alignment with the specified requirements for the main body, title, authors, and affiliations.

**Journal Requirements**

2. As required by our policy on Data Availability, please ensure your manuscript or supplementary information includes the following:

A numbered table of all studies identified in the literature search, including those that were excluded from the analyses. For every excluded study, the table should list the reason(s) for exclusion.

If any of the included studies are unpublished, include a link (URL) to the primary source or detailed information about how the content can be accessed.

A table of all data extracted from the primary research sources for the systematic review and/or meta-analysis. The table must include the following information for each study:

Name of data extractors and date of data extraction

Confirmation that the study was eligible to be included in the review.

All data extracted from each study for the reported systematic review and/or meta-analysis that would be needed to replicate your analyses.

If data or supporting information were obtained from another source (e.g. correspondence with the author of the original research article), please provide the source of data and dates on which the data/information were obtained by your research group.

If applicable for your analysis, a table showing the completed risk of bias and quality/certainty assessments for each study or outcome. Please ensure this is provided for each domain or parameter assessed. For example, if you used the Cochrane risk-of-bias tool for randomized trials, provide answers to each of the signalling questions for each study. If you used GRADE to assess certainty of evidence, provide judgements about each of the quality of evidence factor. This should be provided for each outcome.

An explanation of how missing data were handled.

This information can be included in the main text, supplementary information, or relevant data repository. Please note that providing these underlying data is a requirement for publication in this journal, and if these data are not provided your manuscript might be rejected.

**Authors’ response**

We have ensured full compliance with the journal's Data Availability policy and have provided all necessary data in the manuscript and supplementary materials. Specifically:

- A table listing all studies identified in the literature search, including those excluded from the analysis with reasons for exclusion, is included in the supporting information file.
- None of the included studies are unpublished, and all primary research sources are publicly available.
- The data extracted from primary study sources for the systematic review are provided in the supporting file, detailing all relevant information required to replicate our analyses. These details include names of data extractors and the data extraction date; all extracted data used for the systematic review; and sources and dates for any additional data obtained via correspondence with original study authors or other sources.
- Tables 2 and 3 in the manuscript present the Joanna Briggs Institute (JBI) quality assessment, which includes the completed risk of bias and quality/certainty assessments for each study and confirm that studies are eligible for inclusion in the review.
- A detailed explanation of how missing data were addressed is included in the “Methods, Quality appraisal” section of the manuscript.
- All relevant information has been provided in the main text, supplementary materials, or the manuscript itself in accordance with the journal's requirements.

**Journal Requirements**

3. Please note that your Data Availability Statement is currently missing the DOI/accession number of each dataset and a direct link to access each database. If your manuscript is accepted for publication, you will be asked to provide these details on a very short timeline. We therefore suggest that you provide this information now, though we will not hold up the peer review process if you are unable.

**Authors’ response**

We have included each dataset's DOI/accession number and direct links to access each database in the supporting file.

**Journal Requirements**

4. Please include captions for your Supporting Information files at the end of your manuscript, and update any in-text citations to match accordingly. Please see our Supporting Information guidelines for more information: http://journals.plos.org/plosone/s/supporting-information.

**Authors’ response**

We have included the captions for our Supporting Information files and updated any in-text citations to match accordingly.

**REVIEWER 1 COMMENTS**

**Comments from Reviewer #1**

Thank you for the opportunity to read this interesting article. The topic addressed by the authors of this systematic review is of great interest, and therefore, an article aimed at synthesizing the scientific evidence on the possible association between the onset of cognitive decline and childhood health conditions and socioeconomic status is of utmost cultural and scientific relevance.

At line 46, after the introductory section and before addressing the topic of the role of “early-life nutrition” (line 49), it would be appropriate to guide the reader gradually towards the factors investigated in this systematic review. This could be done by introducing the concept of ‘risk factor’ as a useful element in addressing a health issue of such significant impact in terms of burden on individuals, the population, and healthcare systems, especially considering the aging population and the resulting importance of taking preventive action.

**Authors’ response**
 
Thank you for the helpful suggestion. In response, we have revised the manuscript to better guide the reader towards the factors investigated in this systematic review. We introduced the concept of 'risk factor' after the introductory section, before discussing early-life nutrition (Introduction, lines 47-50):

“*Consequently, understanding causation is crucial to underpin targeted early interventions and diagnoses. It can inform policy and healthcare strategies that may mitigate the significant burden of dementia, and reinforce the disease as a public health priority.*”

**Comments from Reviewer #1**

In the first part of the article, it might be appropriate to include epidemiological information and data on the frequency associated with the factors investigated, which would be useful for framing and sizing the phenomenon.

**Authors’ response**

Thank you for the suggestion. We have incorporated relevant epidemiological information and data on the frequency associated with the factors investigated (Introduction, lines 60-66):

*“Malnutrition affects over 149 million children under five and has significant long-term neurodevelopmental consequences [5]. Over 356 million children are living in extreme poverty and lacking access to basic healthcare, food, education, and resources [6]. There is evidence linking poor health or adverse socioeconomic conditions in childhood with later-stage cognitive decline, suggesting those from poorer backgrounds had a 1.5 to 2.0 times higher risk of cognitive decline and dementia [7].”*

**Comments from Reviewer #1**

It would be useful to contextualize the work conducted by placing it within the framework of risk factor characterization and in relation to prevention. In recent years, there has been a significant production of scientific evidence regarding risk factors for dementia and cognitive decline. Specifically, to date, 14 known risk factors for dementia have been identified (12 at the time indicated in the manuscript), starting from early life (such as low education). In addition, prevention strategies to counter the risk stemming from exposure to these risk factors have been identified and described, including in internationally relevant documents and WHO guidelines. Although the central topic of this review does not concern the known risk factors for dementia, including a paragraph that describes the literature and reference documents on this subject would provide a way to relate the work of this systematic review to what is happening in the field of dementia prevention. This topic could be revisited in the discussion, emphasizing the general need to act preventively from the earliest stages of life, in line with population-wide interventions.

**Authors’ response**

Thank you for the suggestion. We have included scientific evidence related to risk factors for dementia and cognitive impairment, along with references to WHO guidelines, to contextualize our work within the broader framework of dementia prevention (Introduction, lines 51-59).

*“The Lancet Commission on Dementia Prevention, Intervention, and Care identified 14 modifiable risk factors, including childhood-related factors such as low education, which account for 40% of global dementia cases [3]. Lifecourse preventative strategies are supported by the World Health Organization (WHO) 2019 report, “Reducing the Risk of Cognitive Decline and Dementia” [4]. Their guidelines highlight the impact of early-life interventions in mitigating cognitive decline and reinforce a need to evaluate childhood health and socioeconomic conditions as potential contributors to later-life cognitive decline. Thus, this systematic review seeks to reveal early-life risk factors linked to cognitive impairment and dementia.”*

**Comments from Reviewer #1**

In describing the purpose of the systematic review, the objectives through which the authors aim to achieve it could be presented more precisely. Specifically, in the first objective, the factors on which the search string was built could be listed in greater detail. In the second objective, it might be preferable to refer to the 'possible association between the factors described in the first objective and the risk of cognitive decline' rather than 'potential factors.' In any case, this comment is merely a suggestion to make the reading clearer and to facilitate the transition to the following methods section, where the search string is provided.

**Authors’ response**

Thank you for your suggestion. We fully agree with you and have revised the research objectives to provide greater clarity and alignment with our search strategy and analysis approach (Introduction, lines 119-124):

*“ 1.	To systematically identify and synthesize evidence on the relationship between childhood health and SES with cognitive impairment and dementia in later life.*

*2.	To explore the possible association between these early-life factors and the risk of cognitive impairment and dementia, thereby informing future cohort analyses and intervention strategies.”*

**Comments from Reviewer #1**

As currently presented, the search string does not allow for proper replicability of the research. It would be advisable to provide the exact search string as it was launched in the databases.

**Authors’ response**

Thank you for your feedback. We have now included the exact search string as it was launched in the databases to ensure proper replicability of the research (Methods, Search strategy, lines 144- 167).

*“•	MEDLINE: ("child* health*"[All Fields] OR "child* experience*"[All Fields] OR "child* illness"[All Fields] OR "child* disease*"[All Fields] OR "child* socioeconomic"[All Fields] OR "parental education"[All Fields] OR "parental occupation"[All Fields] OR "household income"[All Fields]) AND ("cogn* impairment"[All Fields] OR "cogn* decline"[All Fields] OR "cogn* disorder"[All Fields] OR "dementia"[All Fields] OR "Alzheimer*"[All Fields]) AND ("older adults"[All Fields] OR "older age"[All Fields] OR "older people"[All Fields] OR "elderly"[All Fields] OR "aged"[All Fields] OR "aging"[All Fields]), Filters: in the last 10 years, English.*

*•	CiNAHL: childhood health OR childhood health issues OR childhood socioeconomic status OR ( childhood adversity or childhood trauma or adverse childhood experiences ) AND ( cognitive impairment or cognitive dysfunction or cognitively impaired or dementia or Alzheimer) AND ( older people or older adults or elderly or aged ), Filter: Publication Year: 2014-2024, Peer-Reviewed, English Language, Human.*

*•	PsycINFO: Any Field: child* health* OR Any Field: child* experience* OR Any Field: child* illness OR Any Field: child* disease* OR Any Field: child* socioeconomic OR Any Field: parental education OR Any Field: parental occupation OR Any Field: household income AND Any Field: cogn* impairment OR Any Field: cogn* decline OR Any Field: dementia OR Any Field: Alzheimer* AND Population Group: Human AND Age Group: Middle Age (40-64 yrs) OR Aged (65 yrs & older) AND Document Type: Journal Article AND Open Access AND Peer-Reviewed Journals only AND Year: 2014 To 2024.”*

**Comments from Reviewer #1**

Just before the figure related to the eligibility and exclusion criteria, a sentence summarizing the criteria adopted could be included to facilitate reading.

**Authors’ response**

Thank you for your suggestion. To improve readability and facilitate understanding of the eligibility and exclusion criteria, we have included the following summary before the corresponding figure (Methods, Design, lines 131-134):

*“Briefly, this review focused on studies examining the impact of childhood health and SES on cognitive decline and dementia in older adults, including peer-reviewed research published in the past decade (2014–2024) to ensure the information is up to date.”*

**Comments from Reviewer #1**

In the flowchart shown in figure 1, related to the identification procedure, the number of studies identified for each database should be provided, in addition to the total number of records. The same should be done in the text, just before the figure. Additionally, the authors could specify the type of the 19 studies identified for data extraction.

**Authors’ response**

Thank you for your valuable feedback. We have updated the flowchart in Figure 1 to include the number of studies identified for each database, along with the total number of records. Additionally, we revised the text preceding the figure to reflect this information. Furthermore, we have specified the types of the 44 studies included for data extraction, categorizing them as either cross-sectional or cohort studies.

**Comments from Reviewer #1**

The number of records obtained seems, perhaps, too limited (likely due to the particularly rigid search string). This led me to wonder whether the authors had considered more comprehensive alternatives to the search string used, in order to include a larger number of records and avoid potential selection bias.

**Authors’ response**

Thank you for highlighting this important concern. In response, we expanded the search terms related to childhood socioeconomic status (SES) to include additional variables, such as median household income, parental education level, and parental occupational status. This adjustment has allowed us to identify a broader range of relevant articles for the systematic review. As a result, the total number of records has increased from 1,490 in the previous manuscript to 7,640 in the revised search.

**Comments from Reviewer #1**

In the section on main results (lines 196–199), it would be appropriate to provide more detailed and complete information on the study's findings, particularly regarding confounding factors. In other words, this section could be more interesting if developed in a more exhaustive manner, becoming a general introduction to the following sections; otherwise, it could be removed.

**Authors’ response**

Thank you for your insightful suggestion. After careful consideration and discussion, we decided to remove that section to avoid redundancy and improve the overall structure of the manuscript.

**Comments from Reviewer #1**

The sections "Incidence of dementia" and "Cognitive function scores" are very interesting. However, as currently presented, they do not seem to have any connection with the objective of this review. For this reason, in relation to the following section, it would be appropriate to present the data on dementia incidence (as done by the authors) while also including the main information from the studies cited in this section. For example, the objectives of the cited studies could be provided, along with details about the populations studied (clearly referring to the table where data extracted from the analyzed studies is reported).

The following sentence serves as an example to better explain this suggestion: the study “…”, whose objective was “…”, reported an incidence of “…” in a population of subjects with “…”.

**Authors’ response**

Thank you for your thoughtful feedback. After careful consideration, we have omitted the section "Incidence of dementia and cognitive function scores" as they do not align closely with the objectives of this review.

**Comments from Reviewer #1**

The discussion presents a brief list of considerations on the results collected in this systematic review. This section, in itself, seems more like an extension of the results rather than a proper discussion. The variables examined in this systematic review are extremely interesting and of great relevance to the scientific community focused on the prevention of cognitive decline and dementia. In my opinion, it would be important to further develop the discussion, aiming to create a link with current scientific evidence, guidelines, and international documents on dementia prevention, risk factors, and the need to address these through population-level strategies that also take into account the factors investigated in this work. In summary, the authors should discuss the public health implications of this study, as it contributes to the existing body of evidence, suggesting further elements for debate on risk factors, social determinants of health, and strategies to be defined and implemented. This concept is rightly touched upon in the conclusions but should be developed starting from the discussion.

**Authors’ response**

Thank you for your insightful comment. We appreciate your suggestion to further strengthen the discussion by linking our findings with current scientific evidence and guidelines on dementia prevention. We have incorporated these elements into the revised discussion, highlighting the public health implications of our study and emphasizing the need for population-level strategies that address the identified risk factors and social determinants of health. We hope these revisions align with your expectations and contribute to the ongoing debate in this field (Discussion, lines 494-505):
*“This study synthesizes global evidence, emphasizing the importance of early-life interventions in preventing cognitive decline. Public health initiatives should prioritize enhancing children's physical and mental health while addressing socioeconomic inequalities. These strategies not only benefit individuals but also alleviate the societal and economic burden of cognitive decline and dementia, aligning with WHO guidelines [4]. Beyond the scope of these guidelines, this study shows that promoting reading habits from an early age may serve as a potential protective factor for cognitive health, supporting existing theories on cognitive reserve. Future research is needed to clarify the causal relationship between reading habits and cognitive development. Additionally, a deeper exploration of the impact of childhood infectious diseases on later-life cognition is warranted to inform future preventive strategies.”*

**Comments from Reviewer #1**

Although the acronyms are mentioned in the text, it would be advisable to spell them out in the table (particularly in Table 4) by including the acronym and its corresponding meaning at the end of the table.

**Authors’ response**

Thank you for your suggestion. We have updated Table 4 to include the full spelling of all acronyms, along with their corresponding meanings, at the end of the table.

**Comments from Reviewer #1**

In Table 2, it would be appropriate to include the name of the first author and the year, in addition to the title of the article. The score obtained from each study could, alternatively, be included in Table 4 by adding a column. This would make the table self-explanatory regarding the quality of the studies as well.

**Authors’ response**

Thank you for your suggestion. We have updated Table 2 to include the name of the first author and the year, along with the title of the article, to enhance clarity. However, we have decided to keep the quality scores in both Table 2 and Table 3. This is because Table 4 now contains extensive information related to data extraction.

**Comments from Reviewer #1**

The flow diagram figure appears to be of low graphical quality and, for this reason, is very difficult to read. In general, as reported in the comment R#1. The number of studies identified for each database should be provided, in addition to the total number of records.

**Authors’ response**

Thank you for pointing this out. We have improved the graphical quality of the flow diagram figure to enhance its readability. Additionally, we have updated the figure to include the number of studies identified for each database, along with the total number of records, as suggested.

**REVIEWER 2 COMMENTS**

**Comments from Reviewer #2**

Thank you for the opportunity to review this interesting systematic review of early life factors related to cognitive impairment. Here, the authors reviewed three large databases to identify 1,505 records and eventually include 19 journal articles linking childhood health and SES to dementia and/or cognitive impairment. While I believe the line of inquiry is timely and important, the paper in its current form suffers from important limitations that I believe if the authors can address, will be more likely to contribute meaningfully to this area of literature.

Please see below for my detailed comments that I hope will help to improve the clarity and impact of the manuscript.

1. Search is out of date (completed one year ago) – consider rerunning the search to see if any additional papers would be captured.
**Authors’ response**

Thank you for highlighting this important point. We have updated the manuscript by rerunning the search to capture the most recent research on the topic and have incorporated any newly identified studies.

**Comments from Reviewer #2**

2. I was initially surprised that only 19 papers were included given the broad exposure topic of childhood health and SES. I believe this is because the only search term for ‘childhood soceioeconomic* may miss important variables related to childhood health, including those you have extracted from these papers, such as childhood ACEs, parental involvement, parental income or occupation, childhood reading habits and social interactions, and education. By not explicitly searching the databases for these factors, but rather only extracting them from the studies that been labelled ‘socioeconomic’ factors, you may be missing key areas of literature that may bias your assessment and synthesis presented here. Consider either a) rerunning your search to include as many of these terms as possible; b) re-writing your methods to be clear of your pragmatism in this regard; c) remove childhood SES as a factor of interest for this review.

**Authors’ response**

Thank you for highlighting this important concern as well. In response, we expanded the search terms related to childhood socioeconomic status (SES) to include additional variables, such as median household income, parental education level, and parental occupational status. This adjustment has allowed us to identify a broader range of relevant articles for the systematic review. As a result, the total number of records has increased from 1,490 in the previous manuscript to 7,640 in the revised search.
**Comments from Reviewer #2**

3. It is noted that data from the same studies were included in the review via multiple journal articles (i.e. CHARLS, JAGES) please consider including this as a limitation and explain how it may bias the results of the review.

**Authors’ response**

Thank you for the feedback. We have addressed this point by including it as a limitation and providing an explanation of how it may potentially bias the results (Discussion, Page 44, second paragraph).

**Comments from Reviewer #2**

4. There is a lack of explanation on what ‘childhood health’ in this context entails. Please explain in more detail what measurements were used, what variables they included, and whether they were similar across studies.

**Authors’ response**

Thank you for your comment. We have addressed this point by providing a detailed explanation of what “childhood health” and “childhood SES” entail in this context. Specifically, we have clarified the measurements used and the variables included and noted any similarities or differences across the studies. This information has been incorporated into the manuscript for greater clarity. (Introduction, lines 97-113)

*“Childhood health refers to the physical, mental, and emotional well-being of children from birth through adolescence [18]. In the context of this study, childhood health is defined in various ways, ranging from general assessments to more specific measurements. Most studies use retrospective self-reports, asking participants to rate their childhood health from excellent to poor. Additionally, childhood health can be assessed through factors like hunger or food insecurity, as well as the occurrence of illnesses, such as infections or psychological issues, during childhood.*

*Childhood SES refers to the economic and social conditions experienced during early life, often assessed through a combination of self-reported and objective measures [19]. Common measurements include retrospective self-assessments of social status during childhood, typically using a Likert scale (e.g., high, middle, low), as well as parental education and occupation, which are key indicators of a household's socioeconomic position. Additional variables include household income, financial stability, and community factors, such as the percentage of adults with higher education or in professional occupations. These measures are typically categorized into "high," "middle," or "low" SES and are consistent across studies, although specific variables may vary slightly.”*

**Comments from Reviewer #2**

Paragraph 2: “the bulk of research” – very vague, consider revising.

**Authors’ response**

Thank you for pointing this out. We have removed the phrase "the bulk of research".
**Comments from Reviewer #2**

- Paragraph 2: a rationale/link between childhood illness and SES would be useful and improve rationale.

**Authors’ response**

Thank you for your suggestion. We have incorporated a rationale linking childhood illness and SES (Introduction, lines 71-78):

*“Poor living conditions and limited access to healthcare in children from lower socioeconomic backgrounds mean they are more likely to suffer repeated illnesses [11]. As socioeconomic status (SES) increases, so does access to education and stimulation. Increasingly educated parents provide more learning resources, healthier environments, and greater cognitive stimulation at home, which are linked to improved cognitive function and a reduced risk of dementia in later life [12]. Thus, we can establish that childhood health and SES are factors that pose a long-lasting impact on cognitive health and neurodevelopment.”*
**Comments from Reviewer #2**

Please include quality indicators (e.g. low, moderate, high) for the JBI tool and what threshold would be used to exclude articles from synthesis.

**Authors’ response**

Thank you for your suggestion. We have included low-, mid-, and high-quality indicators for the JBI tool, and only high-quality articles were included in this review (Method, Quality appraisal, lines 195-198).
*“Responses were categorized as "yes" (1 point) and others as 0 points ("no," "unclear," or "not applicable"). Tertile classifications of high- (score >70%), mid- (score 50–70%), and low-quality (score <50%) were assigned using the appropriate JBI checklist. Only “high-quality” papers were included in the review.”*
**Comments from Reviewer #2**

The methods and results sections overlap a bit too much. For instance, the data extraction should be in the methods. Consider revising for clarity and conciseness.

**Authors’ response**

Thank you for your suggestion. We have moved the data extraction section to the Methods to improve the clarity and structure of the manuscript, as recommended.
**Comments from Reviewer #2**

Figure 1 blurry and hard to read.

**Authors’ response**

Thank you for pointing this out. We have improved the graphical quality of Figure 1 to enhance its readability.
**Comments from Reviewer #2**

Records excluded (even at title and abstract stage) should include a reason for exclusion.

**Authors’ response**

Thank you for your feedback. We have created a table detailing the reasons for exclusion at all stages, and this table has been included in the revised supplementary materials.

**Comments from Reviewer #2**

Consider including a rationale for why 2013 was chosen as the start date.

**Authors’ response**

Thank you for your suggestion. Initially, we chose the date range 2013 to 2023 to include the most recent 10 years of research, as the review began in 2023. However, we have now updated the date range to 2014 to 2024 and have explained this choice (Methods, Design section, lines 131-134):

*“Briefly, this review focused on studies examining the impact of childhood health and SES on cognitive decline and dementia in older adults, including peer-reviewed research published in the past decade (2014–2024) to ensure the information is up to date.”*
**Comments from Reviewer #2**

Tables 2 and 3: please include the questions or a legend to the questions (top row) for quality assessment. At present it is unclear what the studies have been scored against. Please also include references for each of the studies in the first column.

**Authors’ response**

Thank you for your suggestion. We have added the quality assessment questions below both Tables 2 and 3 to clarify what the studies have been scored against. We also have included references for each study for better transparency and clarity.
**Comments from Reviewer #2**

Paragraph 2: Consider explaining how reliability was calculated and what it means in this context.

**Authors’ response**

Thank you for your suggestion. In the previous draft, we assessed the reliability of the included articles by scoring them with the JBI tool and calculating the total scores for two study types: cross-sectional and cohort studies. However, this approach seemed unclear. In response to your feedback, we have revised the manuscript to include quality indicators (high, medium, and low) based on the JBI score for each article: high quality (score >70%), medium quality (score 50–70%), and low quality (score <50%). Only high-quality articles (score >70%) were included in this review to ensure reliability.

**Comments from Reviewer #2**

Paragraph 2: This section is very vague.. consider revising:

“While acknowledging potential confounders, many articles offer strategies to address biases, such as handling missing data and using analytical models. Despite focusing on childhood experiences, these factors remain relevant for future investigations”. I’m not sure what this means.

**Authors’ response**

Thank you for your feedback. We completely agree with your suggestion. This section aims to highlight that the studies included in this review rely on retrospective data about childhood, which may introduce bias. However, most of them employed advanced statistical methods to address these biases, ensuring that the studies were still assessed as high-quality. We have revised this section to clarify this point (Results, Study characteristics, lines 300-303):
*“Moreover, most studies relied on retrospective information about childhood health and childhood SES, which may be subject to recall bias and/or missing data. To mitigate these biases, a range of advanced statistical techniques such as structural equation modeling (SEM) [63, 66], latent growth curve modeling [49], sensitivity analysis [36], and path analysis [61] were used.”*

**Comments from Reviewer #2**“Importantly, data collection occurred pre-Covid-19, ensuring its integrity”. Integrity with regards to what?

**Authors’ response**

Thank you for your comment. The purpose of this sentence was to highlight that studies collecting data during the Covid-19 period might have faced interruptions, whereas the studies included in this review collected data before the Covid-19 pandemic, ensuring data completeness. However, we agree with your point that including this information may not be entirely appropriate, and therefore, we have removed this sentence from the revised manuscript.

**Comments from Reviewer #2**

“Studies utilised meticulously crafted questionnaires distributed through national surveys in multiple countries, ensuring rigorous testing”. What does rigor constitute in this context?

**Authors’ response**

Thank you for your feedback. The purpose of this sentence was to convey that these questionnaires underwent thorough piloting and evaluation as they were designed for use at national or even multinational levels. However, we agree that the sentence is somewhat ambiguous in this context. We have revised it in the updated manuscript for clarity (Results, Study characteristics, line 271-286):
*“A number of pre-validated measurement instruments were applied across the evidence. For cognitive function and dementia diagnosis, most studies utilized the Mini-Mental State Examination (MMSE) [35, 36, 38, 42, 44, 51, 55, 56, 59]. MMSE is a validated tool for assessing cognitive function in community settings due to its quick administration, ease of use, and robust validation across diverse populations [70]. The MMSE’s 11 items evaluate five domains of cognitive function: orientation, registration, attention, recall, and language, providing a comprehensive overview of an individual’s cognitive status. This makes it particularly suitable for large-scale surveys and effective screening of cognitive impairment in older populations [70]. Other tools included the Cognitive Abilities Screening Instrument (CASI) [68], Community Screening Interview for Dementia (CSI ‘D’) [58], Cross-Cultural Cognitive Examination (CCCE) [57], Delayed Word Recall Test (DWRT) [44, 63, 65, 66], Geriatric Mental State (GMS) [39], ICD-8, 9, & 10 [54], medical records [41, 60], Quick Mild Cognitive Impairment (QMCI) [30], Telephone Interview for Cognitive Status (TICS) [37, 45, 62], Montreal Cognitive Assessment (MoCA-SA) [26], and Wechsler Adult Intelligence Scale (WAIS) [69].”*
**Comments from Reviewer #2**

The paragraph ‘main findings’ is confusing, please revise for clarity or consider removing

**Authors’ response**

Thank you for your insightful suggestion. After careful consideration and discussion, we decided to omit the “Main findings” section to avoid redundancy and improve the overall structure of the manuscript.

**Comments from Reviewer #2**

The results sections on incidence of dementia and cognitive function scores are detailing findings that were not a clear objective of the study. Consider adding this objective or removing these results.

**Authors’ response**

Thank you for your feedback. After thorough discussion, we also decided to remove the section "Incidence of dementia and cognitive function scores" as they do not align closely with the objectives of this review.

**Comments from Reviewer #2**

Discussion: Include broader implications in discussion. What value added does this study bring?

**Authors’ response**

Thank you for your comment. We have addressed this point by expanding the discussion to include the broader implications of our findings (Discussion, lines 494-505).

*“This study synthesizes global evidence, emphasizing the importance of early-life interventions in preventing cognitive decline. Public health initiatives should prioritize enhancing children's physical and mental health while addressing socioeconomic inequalities. These strategies not only benefit individuals but also alleviate the societal and economic burden of cognitive decline and dementia, aligning with WHO guidelines [4]. Beyond the scope of these guidelines, this study shows that promoting reading habits from an early age may serve as a potential protective factor for cognitive health, supporting existing theories on cognitive reserve. Future research is needed to clarify the causal relationship between reading habits and cognitive development. Additionally, a deeper exploration of the impact of childhood infectious diseases on later-life cognition is warranted to inform future preventive strategies.”*

**REVIEWER 3 COMMENTS**

**Comments from Reviewer #3**

This manuscript presented a systematic review of studies examining surveys of aging and dementia versus childhood trauma and low SES. Mixed reviews correlations have been found in prior work. Thus the goal of this paper was to determine the strength of evidence in relation to different early childhood factors. Systematic review of several databases followed by handsearching and removing duplicates revealed 19 sources that examined the research questions.

This review, if original to this field, could represent an important contribution. I am unsure whether there are other related or similar reviews, as this was not mentioned (though it should be stated explicitly).

**Authors’ response**

Thank you for your detailed feedback. We completely agree with your comment and have included this information in the revised manuscript (Introduction, lines 87-95):

*“In the last few decades, only one systematic review and meta-analysis on early-life factors associated with dementia and cognitive impairment in later life has been conducted [17]. However, epidemiological data in this study spanned from 1865 to 2017, perhaps lacking temporal relevance to today’s society. Search strings were slightly limited for systematic and relevant evidence retrieval, and researcher funding potentially led to intrinsic bias.”*

**Comments from Reviewer #3**

The analysis is presented topically (types of studies, study quality, demographics/tools, dementia incidence, cognitive function, childhood health, educational attainment, SES, childhood 'experiences', reading and 'social habits'). While the facts about these sources are laid out, there seems to be a lack of critical analysis of the sources or synthesis of information. The problems begin in this area. While there is a section called quality analysis, there does not appear to be any discussion of study quality in the manuscript.

**Authors’ response**

Thank you for your feedback. We have revised the "Quality Appraisal" section to provide a clearer of the quality of the articles included in this review (Methods, Quality appraisal, lines 191-201):

*“To assess article quality for this literature review, we utilized the Joanna Briggs Institute (JBI) tool, known for its superior reliability compared to the Critical Appraisal Checklists Programme (CASP) [24]. The JBI checklist comprises eight questions for cross-sectional studies and 11 questions for cohort studies (See Tables 2 and 3 for results of quality appraisal) [25]. Responses were categorized as "yes" (1 point) and others as 0 points ("no," "unclear," or "not applicable"). Tertile classifications of high- (score >70%), mid- (score 50–70%), and low-quality (score <50%) were assigned using the appropriate JBI checklist. Only “high-quality” papers were included in the review. Quality assessments of each study were conducted independently by TL and AM, with cross-checking by AL to minimize bias and ensure that no data were missing in this systematic review”.*

Additionally, we have expanded the discussion on the survey tools used in the reviewed studies to enhance clarity and depth (Results, Study Characteristics, lines 245-270):

*“A number of population databases were noted across the 44 studies. Sixteen studies analyzed data from the Health and Retirement Studies International Family of Studies, including the Health and Retirement Study (HRS) [62, 67], the China Health and Retirement Longitudinal Study (CHARLS) [45-51], the Indonesia Family Life Survey (IFLS) [37], the English Longitudinal Study of Aging (ELSA) [61], the Survey of Health, Aging, and Retirement in Europe (SHARE) [52, 53], the Mexican Health and Aging Study (MHAS) [57], the Irish Longitudinal Study on Ageing (TILDA) [36], and the Longitudinal Ageing Study in India (LASI) [35]. Using these validated population datasets offers several strengths, such as harmonized data collection, diverse cross-cultural samples, and longitudinal assessments of cognitive function. Longitudinal data tracking cognitive changes over time allows for in-depth analyses of the long-term impact of childhood experiences. However, a notable limitation of this methodology is reliance on retrospective self-reports of childhood health and SES and associated recall bias.*

*Other sources of data across the studies were retrieved from surveys, such as the Behavioral Risk Factor Surveillance System Survey (BRFSS) [27, 32, 40], the Japan Gerontological Evaluation Study (JAGES) [31, 55, 56], and the Wisconsin Longitudinal Study (WLS) [66, 69]. Each survey drew from prevalidated cognitive assessment tools, such as dementia diagnosis, medical records, and MMSE. These tools are highly reliable in identifying dementia-related outcomes. Papers drawn from survey data had smaller study populations when compared with larger longitudinal datasets. This may reduce the generalizability and cross-cultural applicability of the findings, but they offer contextual, country-specific foci.*

*In summary, compared to other studies, those in the HRS family excel at capturing long-term, cross-cultural trends, though they still encounter challenges related to recall bias.”*

**Comments from Reviewer #3**

Bald face facts about study characteristics are listed, but not in an interesting manner as they could be. For example, authors note the countries that the data comes from, but there is not commentary on the regions that appear or not in the data.

**Authors’ response**

Thank you for your comment. We acknowledge your point that the presentation of study characteristics could be more engaging. In response, we have revised the section to include commentary on the notable regional disparities in the data. Additionally, we have rewritten other study characteristics to present them in a more compelling manner (Results, Study characteristics, lines 229-238):

*“Research on the relationship between childhood health, SES, and later-life dementia spans globally. This review identified a notable focus on resource-rich countries. The US published 15 studies [26-29, 32, 33, 40, 43, 63-69], plus a multicentre study with the UK [62]. China published eight studies [44-51], and Japan published four studies [30, 31, 55, 56]. Two studies were conducted across Europe [52, 53], while two others were conducted in the UK [61], including one in Scotland [59]. The remaining countries–Australia [41], Finland [54], France [42], India [35], Indonesia [37], Ireland [36], Malaysia [39], Mexico [57], Nigeria [58], South Africa [34], Sweden [60], and Vietnam [38]–each have only one study. This highlights a diverse but uneven distribution of research.”*

**Comments from Reviewer #3**

Further, there is no explanation of the survey tools used in the reviewed studies. Are readers assumed to understand these? I'm not familiar myself, but I would have found this type of source useful should this type of info been explained.

**Authors’ response**

Thank you for your comment. Within the scope of this study, we have explained the most commonly used survey tool (MMSE) in the reviewed studies to make this information clearer and more accessible for readers (Results, Study characteristics, lines 271-279):

*“A number of pre-validated measurement instruments were applied across the evidence. For cognitive function and dementia diagnosis, most studies utilized the Mini-Mental State Examination (MMSE) [35, 36, 38, 42, 44, 51, 55, 56, 59]. MMSE is a validated tool for assessing cognitive function in community settings due to its quick administration, ease of use, and robust validation across diverse populations [70]. The MMSE’s 11 items evaluate five domains of cognitive function: orientation, registration, attention, recall, and language, providing a comprehensive overview of an individual’s cognitive status. This makes it particularly suitable for large-scale surveys and effective screening of cognitive impairment in older populations [70].”*

**Comments from Reviewer #3**

One of the most incriminating omissions is lack of commentary about the types of questions or sample sizes in these tools. For example, authors note that there were >31k respondents to one survey but not which survey.

**Authors’ response**

Thank you for your comment. We have added information about the sample sizes in the revised manuscript and provided a brief commentary (Results, Study characteristics, lines 241-244):

*“Sample sizes across studies varied significantly, ranging from 121 [29] to 515,013 [61], with the risk that the smaller population studies may lack sufficient power to detect meaningful differences or associations.”*

**Comments from Reviewer #3**

A more important question would be which surveys got higher response rates and what types of respondent biases these represent, which is quite an important issue in survey data.

**Authors’ response**

Thank you for your comment. We have added information about the response rates and the potential biases associated with them (Results, Study characteristics, lines 291-300):
*“Only 11 papers reported response rates [26, 30, 31, 34, 36, 40, 48, 51, 55-57]. The highest response rate (91.8%) was observed in the MHAS from the HRS family of surveys [57], whereas the lowest response rate (35.3%) was in a Japanese cross-sectional study [30]. This is notable from a methodological perspective, as higher response rates may be subject to response and selection bias. Individuals with better cognitive function, higher SES, or greater engagement with healthcare systems are more likely to participate, therefore overrepresenting the healthier populations. Recognizing these biases is essential when interpreting results, particularly in studies evaluating datasets that measure early-life factors against later-stage cognitive outcomes.”*

**Comments from Reviewer #3**

There is no direct comparison of the type of data presented in sources, aside from the listing of data from these sources. Further, within the results section, it starts to become unclear which of the sources are represented in the types of data for specific subsections. There is also inconsistent formatting in source description, citation and statistics in these subsections of the results.

**Authors’ response**

Thank you for your valuable feedback. We acknowledge the need for clearer comparison and organization of the data from various sources. In response, we have restructured the results section to provide a more transparent comparison of the data types across sources. Additionally, we have standardized the formatting of source descriptions, citations, and statistical information to improve consistency throughout the results section. We include a brief excerpt to illustrate these changes (Results, Childhood health, lines 324-343):

*“Several studies identified links between childhood health and cognitive impairment or dementia later in life. Sha et al.’s longitudinal study of 10,533 participants linked better childhood health with 1.1 times higher cognitive performance in middle and older age when compared with those reporting poorer childhood health [49]. Cross-sectional studies report even higher likelihood estimates of 1.4–1.74 across India [35] and the US [26]. Similarly, Kobayashi et al.’s population-based analysis of older people linked poor childhood health history to a 28% reduction in cognitive scores later in life [34]. Donley et al.’s Finnish longitudinal study of 2,682 participants revealed that childhood stress approximately doubled the risk of dementia [54]. Findings related to childhood nutrition were also noteworthy. Momtaz’s (2015) Malaysian cross-sectional study of 2,745 participants revealed that childhood food scarcity nearly doubled the likelihood of cognitive impairment in old age [39]. This finding was slightly higher than those reported in studies conducted in Vietnam [38] and China [48], which linked severe childhood hunger to a 1.5 times increased risk of poor cognitive outcomes. Conversely, analysis of data from the Irish longitudinal study on aging linked improved cognitive functions with childhood infectious diseases. They found the more infectious diseases experienced by children, the better their cognitive function later in life. This study of 2,994 participants posits that each additional infectious disease may be associated with a 0.18-point improvement in cognitive functioning [36].”*

**Comments from Reviewer #3**

For improvement of this paper, I would like to see the authors synthesis and analyze the sources critically. What are the strengths and weaknesses of each source individually, plus in comparison? Which sources seem to show evidence against childhood effects or even weaker effects? There are a few smaller issues, but these issues seem fairly substantial so significant work seems needed for improvement.

**Authors’ response**

Thank you for your valuable feedback. We have carefully considered your suggestions and have revised the manuscript to strengthen the critical analysis and synthesis of the included studies. While a detailed discussion of the strengths and weaknesses of all 44 studies is beyond the scope of this review, we have highlighted key methodological considerations, particularly in the Results section (Results, Study characteristics, lines 245-270):

*“A number of population databases were noted across the 44 studies. Sixteen studies analyzed data from the Health and Retirement Studies International Family of Studies, including the Health and Retirement Study (HRS) [62, 67], the China Health and Retirement Longitudinal Study (CHARLS) [45-51], the Indonesia Family Life Survey (IFLS) [37], the English Longitudinal Study of Aging (ELSA) [61], the Survey of Health, Aging, and Retirement in Europe (SHARE) [52, 53], the Mexican Health and Aging Study (MHAS) [57], the Irish Longitudinal Study on Ageing (TILDA) [36], and the Longitudinal Ageing Study in India (LASI) [35]. Using these validated population datasets offers several strengths, such as harmonized data collection, diverse cross-cultural samples, and longitudinal assessments of cognitive function. Longitudinal data tracking cognitive changes over time allows for in-depth analyses of the long-term impact of childhood experiences. However, a notable limitation of this methodology is reliance on retrospective self-reports of childhood health and SES and associated recall bias.*

*Other sources of data across the studies were retrieved from surveys, such as the Behavioral Risk Factor Surveillance System Survey (BRFSS) [27, 32, 40], the Japan Gerontological Evaluation Study (JAGES) [31, 55, 56], and the Wisconsin Longitudinal Study (WLS) [66, 69]. Each survey drew from prevalidated cognitive assessment tools, such as dementia diagnosis, medical records, and MMSE. These tools are highly reliable in identifying dementia-related outcomes. Papers drawn from survey data had smaller study populations when compared with larger longitudinal datasets. This may reduce the generalizability and cross-cultural applicability of the findings, but they offer contextual, country-specific foci.*

*In summary, compared to other studies, those in the HRS family excel at capturing long-term, cross-cultural trends, though they still encounter challenges related to recall bias.”*

We hope these changes improve the clarity of the manuscript and that you will be satisfied with the revised manuscript.
